# Association of Sleep Quality and General, Mental, and Oral Health with Lifestyle Traits (Dietary Intake, Smoking Status) in Arthritis: A Cross-Sectional Study from the Canadian Community Health Survey (CCHS)

**DOI:** 10.3390/nu16132091

**Published:** 2024-06-29

**Authors:** Zohre Gheisary, Ibrahim Hoja, Juxin Liu, Petros Papagerakis, Lynn P. Weber, Mark Fenton, George S. Katselis, Jessica R. L. Lieffers, Silvana Papagerakis

**Affiliations:** 1 Department of Biochemistry, Microbiology, and Immunology, College of Medicine, University of Saskatchewan, 107 Wiggins Road, Saskatoon, SK S7N 5E5, Canada; zog389@usask.ca; 2 Laboratory of Precision Oral Health and Chronobiology, Faculty of Dentistry, Laval University, Dental Medicine Pavilion, 2420, rue de la Terrasse, Quebec City, QC G1V 0A6, Canada; ibhoj@ulaval.ca (I.H.); petros.papagerakis@fmd.ulaval.ca (P.P.); 3 Health Sciences, College of Medicine, University of Saskatchewan, 107 Wiggins Road, Saskatoon, SK S7N 5E5, Canada; 4 Department of Mathematics and Statistics, College of Arts and Science, University of Saskatchewan, 106 Wiggins Road, Saskatoon, SK S7N 5E6, Canada; jul086@mail.usask.ca; 5 Department of Veterinary Biomedical Sciences, Western College of Veterinary Medicine, University of Saskatchewan, 52 Campus Drive, Saskatoon, SK S7N 5B4, Canada; lynn.weber@usask.ca; 6 Division of Respirology, Critical Care, and Sleep Medicine, College of Medicine, University of Saskatchewan, Saskatoon, SK S7N 5E5, Canada; mef132@mail.usask.ca; 7 Department of Medicine, Canadian Centre for Rural and Agricultural Health, College of Medicine, University of Saskatchewan, 104 Clinic Place, Saskatoon, SK S7N 2Z4, Canada; george.katselis@usask.ca; 8 College of Pharmacy and Nutrition, University of Saskatchewan, 107 Wiggins Road, Saskatoon, SK S7N 5E5, Canada

**Keywords:** arthritis, lifestyle, diet, smoking, sleep, health status, logistic regression

## Abstract

Arthritis is associated with health challenges. Lifestyle traits are believed to influence arthritis development and progression; however, data to support personalized treatment regimens based on holistic lifestyle factors are missing. This study aims to provide a comprehensive list of associations between lifestyle traits and the health status of individuals with arthritis in the Canadian population, using binary logistic regression analysis on data from the Canadian Community Health Survey, which includes 104,359 respondents. Firstly, we explored the association between arthritis and various aspects of health status including self-reported lifestyle factors. Secondly, we examined the associations between self-reported dietary intake and smoking status with general, mental, and oral health, and sleep disturbance among individuals both with and without arthritis. Our analysis revealed that individuals with arthritis reported considerably poorer general, mental, and oral health, and poorer sleep quality compared to those without arthritis. Associations were also found between self-reported dietary intake and various measures of health status in individuals with arthritis. Smoking and exposure to passive smoking were associated not only with arthritis but also with compromised sleep quality and poorer general, mental, and oral health in people with and without arthritis. This study highlights the need for personalized and holistic approaches that may include a combination of dietary interventions, oral health improvements, sleep therapies, and smoking cessation for improved arthritis prevention and care.

## 1. Introduction

According to the latest update from the Arthritis Community Research and Evaluation Unit (ACREU) on the status of arthritis in Canada released in 2019, around six million (~1 in 5) Canadians have been impacted by arthritis [1]; these authors further estimate this number will grow to over 24% by 2040 [2]. Arthritis is a chronic disease that comprises over 100 different diseases affecting joints, surrounding and other connective tissues and is associated with pain, discomfort, major functional disability, and activity restriction [1,2,3]. Moreover, it is associated with poor general health and a higher prevalence of other chronic diseases/comorbidities including cardiovascular diseases, diabetes, cancer, metabolic syndrome, sleep disturbance, and mental health issues including depression, mood disorders, and anxiety [4,5,6,7]. Arthritis is also more prevalent in women, older adults, in individuals who are overweight, and in people who have lower income and/or lower education levels [1].

Studies have also shown that people with arthritis are more likely to report sleep disturbances compared to people without arthritis [8,9,10,11,12,13]. More specifically, individuals with arthritis report various sleep issues including short sleep duration (i.e., less than 6 h per night), daytime sleepiness, difficulty staying awake and going to sleep, non-refreshing sleep, poor to fair quality of sleep, and insomnia [11,13]. Also, the frequency and type of sleep disturbance varies depending on the type of arthritis [13]. In both rheumatoid arthritis (RA) and osteoarthritis (OA), greater sleep disturbance is correlated with disease activity and increased arthritic joints and joint pain [12,14]. Also, sleep problems in people with arthritis are more frequently reported in younger individuals, women, individuals with lower income, and those with a higher body mass index (BMI) [3,15]. Numerous reasons could explain poor sleep outcomes in people with arthritis, including joint pain and discomfort. Also, another possible reason for a relationship between arthritis and sleep quality is the reciprocal connection between sleep and the immune system [16]. Sleep has an important role in regulating the immune system dynamically through affecting immune cells, including antigen-presenting cell and T-cell distributions and the production of inflammatory cytokines [16]. Therefore, sufficient and high-quality sleep is fundamental to maintaining overall health as it plays a critical role in enhancing immune function. Insufficient sleep and untreated sleep disorders have also been shown to be associated with several medical epidemics and health issues, including poorer general health, oral health, and mental health [17,18,19].

Another common health concern in individuals with arthritis is mental wellness. According to the Centers for Disease Control and Prevention (CDC), 22.5% and 12.1% of adults with arthritis report symptoms of anxiety and depression, respectively, compared to 10.7% and 4.7% of adults without arthritis [20]. Also, a multi-national cross-sectional study using eighteen population-based surveys from different countries that assessed the association between arthritis and anxiety and mood disorders found that the prevalence of mental disorders is higher in persons with arthritis compared to persons without arthritis [21]. This result was also confirmed by another muti-national study that investigated the relationship between arthritis and mental health outcomes including depression, anxiety, and stress across 46 low- and middle-income countries. This work also showed a higher frequency of arthritis in women compared to men and a lower frequency of arthritis among individuals with more education [22]. Also, the importance of mental health issues in people with arthritis is not limited to its association with poorer RA outcomes and quality of life; mental health issues have also been found to predict a reduced response to treatment due to poor medication compliance [22,23].

In addition to the mentioned health complications, arthritis is associated with a higher probability of struggling with oral health including a higher prevalence of periodontitis [24,25]. In 2008, Pischon et al. found that the association between RA and periodontitis was independent of demographic and lifestyle factors such as sex and age [26].

In people with arthritis, lifestyle factors (e.g., smoking, physical activity, alcohol use, and diet) may also have a role in the development and progression of the disease. Numerous studies have found a relationship between RA and smoking. For example, a meta-analysis study of 18 case–control and cohort studies in the United States and Europe found that current male and female smokers have a higher risk of developing RA compared to non-smokers. Additionally, the meta-analysis study revealed that heavy smokers, defined as individuals with a smoking history of 20 pack-years or more, have an elevated risk of developing RA regardless of gender [27]. Furthermore, a meta-analysis of six case–control and prospective cohort studies conducted in the United States and Europe reported that individuals exposed to passive smoking have a 12% higher prevalence of RA versus individuals who are not exposed [28].

Several studies have consistently shown that individuals with RA also tend to have lower levels of physical activity compared to those without this issue [29,30]. However, multiple studies have consistently found that exercise and increased physical activity are linked to reduced disease activity and systemic inflammation in individuals with RA [31,32,33]. Furthermore, various studies have provided evidence that exercise interventions in individuals with RA have had a positive impact on disease symptoms, specifically in reducing pain levels [34], alleviating fatigue [35], and enhancing overall functioning [36].

In studies that examined the effect of alcohol consumption on RA, different prospective and cross-sectional studies have suggested that alcohol consumption reduces both the risk of developing RA and the severity of this disease [37,38]. In a study conducted by Maxwell et al., they observed that alcohol consumption was linked to a significant and dose-dependent decrease in susceptibility to RA [39]. Moreover, a prospective study from Sweden found that alcohol consumption was associated with reduced RA disease activity in females but not in males [40]. Moreover, in 2009, Källberg et al. assessed the interaction between alcohol consumption and smoking in influencing RA risk and found that drinking alcohol reduced risk more pronouncedly among smokers than non-smokers [41]. This reduction in risk could be explained by the anti-inflammatory effects associated with moderate alcohol consumption [42,43].

Diet is another lifestyle factor that has been shown to have an impact on RA, including the development and progression of this disease. High-fat diets (HFDs) are an important factor leading to obesity [44]. This relationship is important as a meta-analysis study concluded that obesity was associated with a higher disease activity score 28 in people with RA [45]. A meta-analysis of observational studies also found evidence that the association between obesity and an increased risk of RA is stronger among females than males [46]. Moreover, the intake of high-fructose beverages at least five times a week increases the risk of developing arthritis [47]. In 2014, Hu et al. also found that the consumption of sugar-sweetened soda increases the risk of RA among women [48]. On the other hand, certain dietary components could play a role in reducing the risk and activity of RA. For example, a case-control study has demonstrated that the Mediterranean diet may lower the risk of RA among males with seropositive RA [49]. Kostoglou-Athanassiou et al. have also suggested that omega-3 fatty acids may reduce RA activity [50]. Moreover, in 2018, Tedeschi et al. proposed that consuming fish more than twice a week reduces RA activity [51].

Lifestyle factors not only contribute to the risk and progression of RA but are also linked to the occurrence of sleep disorders. For example, several studies have found that smoking reduces sleep quality, and it is a significant risk factor associated with sleep disorders [52,53,54]. Also, a Japanese study found that women who were current smokers had more difficulty initiating sleep than women who had never smoked [55]. The association of alcohol use with sleep is a complex phenomenon. Many studies have also shown an increased prevalence of insomnia among alcohol-dependent individuals [56,57]. Furthermore, alcohol dependence is associated with obstructive sleep apnea and insufficient sleep duration [58,59]. The association between sleep disorders and alcohol consumption may also vary according to gender and race [60]. Also, physical activity is a lifestyle factor that contributes to improved health and reduces the risk of chronic diseases [61]. Diet is also a lifestyle factor that has a complex relationship with sleep. For example, St-Onge et al. found that a high-carbohydrate intake reduces sleep onset latency and a high-fat intake decreases sleep efficiency [62]. A cross-sectional study also found an association between an unhealthy diet and poor sleep quality [63], and Campanini et al. suggested that following a Mediterranean diet decreases the risk of poor sleep quality [64]. Also, an observational study found that individuals with insomnia reported a higher intake of fat than individuals without sleep disorders [65]. However, a conspicuous knowledge gap persists regarding understanding the association of lifestyle factors with sleep quality in people with arthritis. Closing this scientific gap is imperative for the development of targeted interventions specifically tailored to address sleep disturbances in this population, considering their distinctive lifestyle challenges.

Lifestyle factors also have an important impact on mental health. Various studies have found a strong association between smoking and mental health issues such as anxiety and affective disorders [66,67]. Similarly, individuals consuming higher amounts of alcohol are more likely to have mental health issues, such as anxiety and depression, compared to those who do not [68,69]. Mental health is also affected by physical activity. Physical activity has been shown to have positive psychosocial outcomes and a beneficial effect on reducing anxiety in young people [70]. Dietary behaviors have also been shown to have an influence on mental health. A healthy diet is associated with a reduced risk of mental health issues [71]. Also, a cross-sectional study showed that high fruit and vegetable intake are important for mental and physical health [72]; moreover, diets rich in crucial nutrients such as omega-3 fatty acids and B vitamins have an important role in promoting good mental health [73]. However, there is a notable gap in understanding the association of these lifestyle factors with mental health in individuals with arthritis; addressing this scientific gap helps to better understand and address the intricate relationships between lifestyle choices and mental well-being in people with arthritis.

Given the aforementioned associations, we hypothesize that positive lifestyle factors, such as modifying dietary intake and smoking status, could be positively associated with various measures of health status in people with arthritis. These health status measures include sleep disturbances, mental health issues, general health problems, and oral health issues, which often co-occur with the main symptoms of arthritis (such as joint pain and swelling). To confirm this hypothesis, we used data from the Canadian Community Health Survey (CCHS), which is a large Canadian annual health survey that captures information on chronic diseases, health status, and lifestyle factors in a representative sample of Canadian residents. To the best of our knowledge, this is the first study to examine the associations between lifestyle traits and arthritis and also association of various lifestyle traits on different health status measures in people with and without arthritis in a large sample of Canadians.

**Figure 1:**
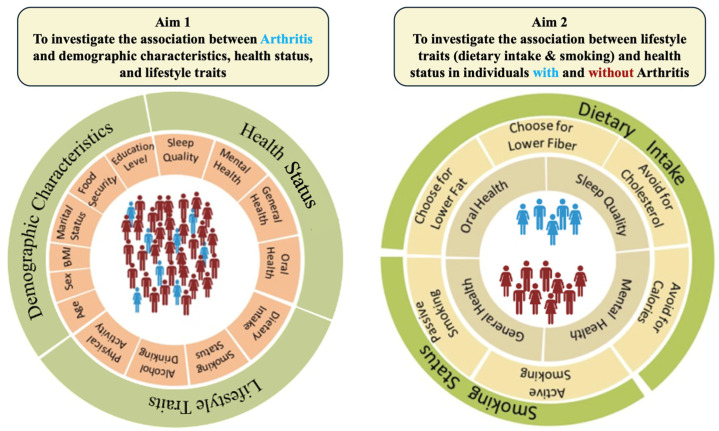
Study design and research question. The study design consists of two primary aims. The first aim of this study is to investigate associations between arthritis and demographic characteristics, health status, and lifestyle traits. The second aim is to investigate the association of lifestyle traits including dietary intake (choose lower fat/higher fiber and avoid cholesterol/calories) and smoking status (active/passive) with health status including sleep quality and general, mental, and oral health in individuals with and without arthritis. “Created with BioRender.com.”.

The current study design consists of two primary aims. The first aim of this study is to a) investigate associations between arthritis and demographic characteristics as well as various aspects of self-reported health status (i.e., sleep quality, general health, mental health, and oral health) and b) investigate associations between arthritis and self-reported lifestyle traits (i.e., dietary intake, smoking status, drinking status, and physical activity). The second aim of this study is to a) assess associations between self-reported lifestyle traits (i.e., dietary intake and smoking status) and health status (i.e., sleep quality and general, mental, and oral health) and b) investigate the associations between sleep quality and duration and health status (i.e., general, mental, and oral health) in individuals with and without arthritis, separately (Figure 1).

## 2. Materials and Methods

### 2.1. Study Design, Data Source and Population

Data for this cross-sectional study were obtained from the 2017–2018 Canadian Community Health Survey (CCHS) public use microdata file. The CCHS is an annual cross-sectional survey with the central objective of gathering information to monitor health status, the use of health care services, and health determinants in the general population of Canadians aged 12 years and older. The 2017–2018 CCHS collected data from approximately 113,000 respondents. This survey provides information on a sample of representative individuals in the ten provinces and three territories of Canada. This survey excluded persons living on reserves and other Aboriginal settlements, youth aged 12–17 years living in foster homes, full-time members of Canadian Forces, institutionalized populations, and persons living in the Quebec health regions of Nunavik and Terres-Cries-de-la-Baie-James, which altogether encompassed less than 3% of the target population. Further details on sampling strategies and questionnaires are described elsewhere [74]. The CCHS microdata file is publicly available and was therefore deemed exempt from review by the University of Saskatchewan Research Ethics Board.

**Figure 2:**
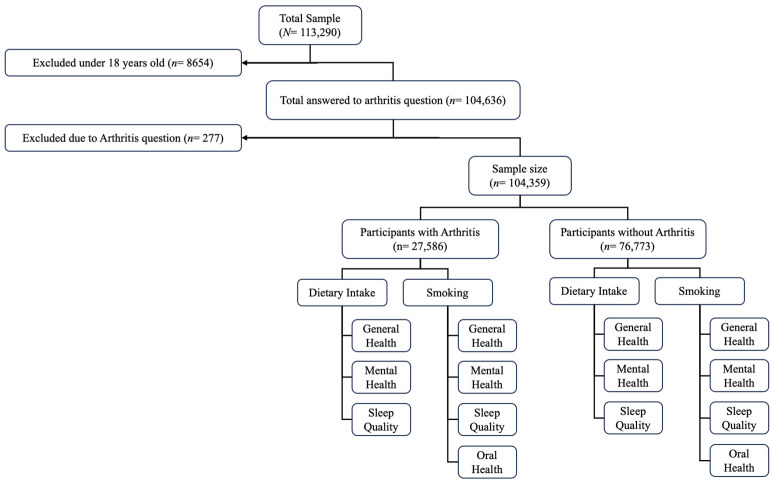
Flow diagram showing the final analytic sample for each outcome from the 2017–2018 CCHS.

In this study, we selected individuals aged 18 years and older who answered a question in the survey regarding arthritis status. Figure 2 shows participant flow.

### 2.2. Measures

#### 2.2.1. Outcome Variables

1. For the first research aim, the outcome of interest was self-reported arthritis status derived from the question: “Do you have arthritis, for example osteoarthritis, rheumatoid arthritis, gout or any other type, excluding fibromyalgia?” (“Yes”, “No”).
2. For the second research aim, we separated the population based on their answer to the question on arthritis status mentioned above. With these two separate groupings (i.e., those who self-reported having arthritis; those who self-reported not having arthritis), we assessed the associations of a) self-reported lifestyle traits (i.e., dietary intake and smoking status) on several health status measures of interest including sleep quality, general health, mental health, and oral health and b) the associations of sleep quality and duration on health status measures of interest including general health, mental health, and oral health. It should be noted that in the second part of the second research question (i.e., 2b), “sleep quality” and “duration” are treated as independent variables, whereas for the first study aim they are considered outcome variables, along with general health, mental health, and oral health. These outcomes are described in more detail below.

Sleep quality: Responses to three questions related to sleep quality were used as separate outcomes. The three questions were as follows: “How often do you find your sleep refreshing?”; “How often do you find it difficult to stay awake when you want to?”; “How often do you have trouble going to sleep or staying asleep?”. Responses for all three questions included “Never”, “Rarely”, “Sometimes”, “Most of the time”, and “All of the time.” Respondents who answered “Never” or “Rarely” were classified as not refreshed, not troubled, and no difficulty sleeping, respectively.

General health, mental health, and oral health: The overall self-reported status of general health, mental health, and oral health was derived from the questions asking “In general, would you say your health/mental health/oral health is...?”. Responses included “Excellent”, “Very Good”, “Good”, “Fair”, and “Poor”. Responses were grouped into the following categories: “Poor” (fair/poor) and “Good” (excellent/very good/good).

Self-reported chronic conditions were also included, including diabetes, cancer, high blood cholesterol, and high blood pressure. The self-reported status of these conditions was derived from the questions asking “Do you have diabetes?”, “Do you have cancer?”, “Do you have high blood pressure?”, and “Do you have high blood cholesterol or lipids?”. Response options included “Yes” and “No” and were grouped into those two categories.

Mental health (i.e., mood and anxiety disorders): Mood and anxiety disorders were derived from the following questions: “Do you have a mood disorder such as depression, bipolar disorder, mania or dysthymia?” and “Do you have an anxiety disorder such as a phobia, obsessive-compulsive disorder or a panic disorder?”. Response options included “Yes” and “No” and were grouped into those two categories.

Oral health: In addition to overall oral health, several self-reported oral health outcomes were also included, including bleeding gums, mouth pain, mouth dryness, and uncomfortable eating. The status of bleeding gums, mouth pain, mouth dryness, and uncomfortable eating were derived from the questions asking “How often have you had bleeding gums while brushing or flossing your teeth?”, “How often have you had any other persistent or ongoing pain anywhere in your mouth?”, “How often have you had persistent dry mouth?”, and “How often have you found it uncomfortable to eat any food because of problems with your mouth?”. Responses included “Often”, “Sometimes”, “Rarely”, and “Never”. Responses were grouped into the following categories: “Yes” (often/sometimes) and “No” (rarely/never).

#### 2.2.2. Independent Variables

Several independent variables of interest were included, which are described in detail below:

Sociodemographic variables: The independent variables of interest for both study aims included respondent self-reported demographic information (sex (male/female); age (years, grouped as follows: 65 years and older, 50–64 years, 35–49 years, and 18–34 years); marital status (married/common law, widowed/divorced/separated, and single); education (less than secondary school graduation, secondary school graduation, and post-secondary certificate diploma or university degree); food security (moderate/severe food insecurity and food secure).

Sleep quality variables: For the first research aim, the sleep variables included as independent variables were the same as listed for the outcome variables described earlier for the second research aim.

General health/mental health/oral health: For the first research aim, the general health, mental health, and oral health variables that were included as independent variables were the same as described for the outcome variables discussed earlier for the second research aim.

Lifestyle variables: Several independent variables related to self-reported lifestyle traits were also included. These variables are described in more detail below.

- Body mass index (BMI): BMI (kg/m^2^) was calculated using self-reported height and weight according to international standard classification: underweight (<18.5); normal weight (18.5–24.9); overweight (25–29.9); obese class I, II, and III (≥30).
- Self-reported food choices: Information on the self-reported intake of fat, fiber, cholesterol, and calories was also included. The following questions were used to derive this information: “Do you choose certain foods because of the lower fat content?” (responses included “Yes” and “No”), “Do you choose certain foods because of the fibre content?” (responses included “Yes” and “No”), “Do you avoid certain foods because of the cholesterol content?” (responses included “Yes” and “No”), and “Do you avoid certain foods because of the calorie content?” (responses included “Yes” and “No”). Responses to each of these questions were grouped into two categories: “Yes” and “No.”
- Fruit and vegetable consumption: The total self-reported daily consumption of fruits and vegetables was categorized as “Eats fruits and vegetables less than 5 times per day”, “Eats fruits and vegetables between 5 and 10 times per day”, and “Eats fruits and vegetables more than 10 times per day.” We recategorized the total fruit and vegetable consumption as “Eats fruits and vegetables less than 5 times per day” and “Eats fruits and vegetables equal or more than 5 times per day.”
- Smoking status: Smoking status was categorized based on the respondents’ self-reported smoking habits. The response options included “Current daily smoker and current occasional smoker”, “Former daily smoker (non-smoker now) and former occasional smoker (non-smoker now)”, “Lifetime abstainer (never smoked a whole cigarette”, and “Experimental smoker (at least 1 cig, non-smoker now).” We recategorized these response options into “Current daily/occasional smoker (current smoker)”, “Former daily/occasional smoker (former smoker)” and “Experimental smoker or lifetime abstainer (never smoker).”
- Exposure to second-hand smoke: Information on self-reported second-hand smoking exposure was also included by using information captured from the following question: “Including both household members and regular visitors, does anyone smoke inside your home, every day or almost every day?” Responses were grouped into two groups: “Yes” and “No”.
- Alcohol use: Self-reported alcohol drinking habits were categorized as “Regular drinkers who drink at least once a month to every day”, “Occasional drinkers who drink less than once a month”, and “Never drinkers who never had a drink or did not drink lifetime or during the past 12 months.”
- Physical activity: According to Canadian Physical Activity Guideline (CPAG), adults are recommended to have at least 150 min of moderate-to-vigorous-intensity aerobic activity per week. Self-reported physical activity was categorized according to the CPAG, which included “Physically active at/above recommended level from CPAG” for individuals with equal to or more than 150 min of activity, “Physically active below recommended level from CPAG” for individuals with less than 150 min of activity, and “No physical activity” for individuals with 0 min of activity reported.

### 2.3. Data Analysis

Descriptive statistics were used to determine the frequencies of the independent variables cross-tabulated with the outcome variables of interest.

For the first research aim, we examined the association between arthritis and each demographic characteristic (sex, age, marital status, education, and food security), self-reported lifestyle trait (i.e., dietary intake, smoking status, drinking status, BMI, and physical activity), and health status measure (general health, mental health, oral health, and sleep quality) using univariate logistic regression. For the second research aim, first we identified the five most important and well-known arthritis confounders amongst the demographic characteristics (age and sex) and lifestyle traits (BMI, smoking status and drinking status). We then conducted univariate logistic regression analysis to assess the crude association between each independent variable of interest (lifestyle traits including dietary intake and smoking status) and the outcome variables (sleep quality, general health, mental health, and oral health) in people with and without arthritis separately (Model Type 1). For Model Type 2, we included demographic characteristics (age and sex). The final model (Model Type 3) included BMI, smoking status and drinking status as covariates in addition to age and sex. The significant contribution of adding BMI, smoking status (where relevant), and drinking status was confirmed by the forward selection based on likelihood ratio tests. The Hosmer Lemeshow goodness-of-fit test was used to assess the goodness of fit with a *p*-value > 0.05 indicating the model is a good fit.

Participants were weighted by the sampling weight provided in the CCHS microdata file allowing for the findings to be attributed to the general Canadian population [75]; these sample weights were applied in all analyses. All analyses were carried out using SPSS software, Version 28.0 (IBM, Armonk, NY, USA).

## 3. Results

### 3.1. Association of Sociodemographic Factors, Lifestyle Traits, and Other Aspects of Health Status with Arthritis

Regression statistics were conducted to examine the association of arthritis with various factors, including sociodemographic factors, lifestyle traits, and other aspects of health status.

#### 3.1.1. Sociodemographic Information

A total of 104,359 participants (54.2% females and 45.8% males) constituted the analytical sample; 27,586 (26.4%) participants self-reported arthritis. In total, females consisted of 61.5% of all participants who self-reported arthritis. Females were 50% more likely to self-report arthritis compared to males (odds ratio (OR) 1.50; 95% confidence interval (CI) 1.46–1.55). The analysis showed that adults ≥65 years of age and 50–64 years of age were 31.09 (95% CI: 28.69–33.70) and 14.57 (95% CI: 13.43–15.80) times more likely to self-report arthritis compared to those who were 18–34 years of age, respectively. Married/common law participants were 1.95 (95% CI: 1.88–2.04) times more likely to self-report arthritis compared to single participants. Moreover, participants with less than a secondary school education were 2.42 (95% CI: 2.34–2.51) times more likely to self-report arthritis compared to those who had post-secondary education degrees. Additionally, we found that individuals experiencing moderate to severe food insecurity were 1.18 (95% CI: 1.12–1.24) times more likely to self-report arthritis when compared to individuals who were food secure. More details information are presented in Table 1.

#### 3.1.2. Lifestyle Traits

Table 2 summarizes the association between self-reported lifestyle traits (such as BMI, food intake, physical activity, alcohol intake, and smoking) and arthritis. Participants who self-reported a BMI that was overweight or obese were 1.45 (95% CI: 1.40–1.50) and 2.11 (95% CI: 2.04–2.19) times more self-report to have arthritis compared to those who were normal weight, respectively. Participants who were not physically active were 2.22 (95% CI: 2.15–2.29) times more likely to self-report arthritis compared to those who had physical activity levels meeting or exceeding the recommended level from CPAG. However, regular alcohol drinkers were 46% less likely to have arthritis compared to those who did not drink.

The number of respondents who answered both questions about arthritis and dietary intake was around 13% of the total population. Our results found there was a statistically significant association between self-reported arthritis and food choice. Participants who choose lower fat foods, choose foods because of fiber content and avoid foods because of cholesterol content were more likely to self-report arthritis compared to individuals who did not follow those behaviors (lower fat content: OR 1.39, 95% CI: 1.28–1.50; fiber content: OR 1.47, 95% CI: 1.36–1.60; avoiding cholesterol content: OR 1.30, 95% CI: 1.20–1.40). However, the association was not statistically significant between arthritis and avoiding calorie content (*p*-value: 0.315). In addition, we found no statistically significant association between self-reported arthritis and fruit and vegetable intake (*p*-value: 0.780).

For smoking, the number of respondents who answered both questions about arthritis and smoking status was 99.6% of the total population. Current and former smokers were more likely to self-report arthritis (current smoker: OR 1.20, 95% CI: 1.15–1.25; former smoker: OR 1.94, 95% CI: 1.88–2.00) compared to never smokers, respectively. Regarding exposure to second-hand smoke, those with exposure to second hand smoke at home were significantly more likely to self-report arthritis compared to those who did not have those exposures (OR 1.28, 95% CI: 1.18–1.40) (Table 2).

#### 3.1.3. Sleep Quality

The analysis of the association between sleep quality and arthritis revealed that participants who found their sleep refreshing had a 32% lower likelihood of self-reporting arthritis compared to those who did not find their sleep refreshing. In addition, participants who experienced difficulty staying awake (OR 1.21, 95% CI: 1.16–1.27) and had trouble going to sleep (OR 1.63, 95% CI: 1.57–1.70) were more likely to self-report arthritis compared to participants who did not report these sleep issues. Furthermore, participants who slept ≥7 h per night were 21% less likely to self-report having arthritis compared to those who slept <7 h per night (OR 0.79, 95% CI: 0.76–0.82) (Table 3).

#### 3.1.4. General Health

We found a significant association between self-reported general health and arthritis. More specifically, we found that the participants with poor general health were more likely to self-report arthritis compared to those with good general health (OR 3.85, 95% CI: 3.71–3.99) (Table 3). Moreover, our findings demonstrated a strong association between chronic health status and the occurrence of arthritis. Participants with chronic health conditions, including diabetes (OR 2.67, 95% CI: 2.56–2.78), cancer (OR 2.29, 95% CI: 2.11–2.49), high total cholesterol (OR 2.75, 95% CI: 2.65–2.85), and high blood pressure (OR 3.28, 95% CI: 3.18–3.38) were more likely to self-report arthritis compared to those without a chronic health condition (Table 3).

#### 3.1.5. Mental Health

When looking at the association between mental health and arthritis, participants with poor mental health were 61% more likely to self-report arthritis (OR 1.61, 95% CI: 1.53–1.69) compared to those self-reporting good mental health (Table 3). Additionally, our findings revealed that individuals who self-reported mood disorders (OR 1.69, 95% CI: 1.62–1.77) and anxiety disorders (OR 1.46, 95% CI: 1.40–1.52) were more likely to self-report arthritis compared to those without mood disorders and anxiety disorders, respectively (Table 3).

#### 3.1.6. Oral Health

Our results identified that the participants with poor oral health were more likely to self-report arthritis compared to those with good oral health (OR 1.59, 95% CI: 1.50–1.69). Furthermore, our findings revealed that participants who self-reported mouth pain (OR 1.46, 95% CI: 1.38–1.55) and mouth dryness (OR 2.70, 95% CI: 2.58–2.82) were more likely to self-report arthritis compared to those without mouth pain and mouth dryness, respectively. Moreover, we found that the participants who felt uncomfortable eating (OR 1.52, 95% CI: 1.44–1.59) were more likely to self-report arthritis compared to those who did not. On the other hand, the participants who had bleeding gums were 30% less likely to self-report arthritis compared to those who had healthy gums (Table 3).

### 3.2. The Association between Lifestyle Traits and Health Status in Participants with and without Arthritis

A binary logistic regression analysis was used to generate different models to examine the association between lifestyle traits (i.e., various self-reported dietary intake behaviors and smoking) and health status (sleep quality, general health, mental health, and oral health) in two different groups (i.e., (a) people with arthritis and (b) people without arthritis). Three logistic regression models were run for each association:

- Model 1 examined the (crude) unadjusted association;
- Model 2 examined the association, while adjusting for the effects of age and sex;
- Model 3 examined the association, while adjusting for age, sex, BMI, smoking status (where relevant) and drinking status.

Table 4 and Table 5 provide the association information (OR) for each relationship for Model 3; this information will also be described in the following sections. Information about the associations for Models 1 and 2 is provided in Supplementary Materials (Tables S1 and S2).

#### 3.2.1. Self-Reported Health Status and Dietary Intake Behaviors

##### Sleep Quality and Dietary Intake Behaviors

The number of respondents with arthritis who answered questions about self-reported dietary intake behaviors (i.e., choosing lower fat foods, choosing high fiber foods, avoiding high cholesterol foods, and avoiding high calorie foods) and sleep quality (i.e., refreshing sleep, difficulty staying wake, trouble going to sleep, and sleep duration) was around 12% of the sample who reported having arthritis. The number of respondents without arthritis who met these criteria was around 13% of the sample without arthritis (Table 4).

When examining the relationship between sleep quality and self-reported dietary behaviors while adjusting for age, sex, BMI, and smoking and drinking status, our results showed that participants with arthritis who self-reported choosing foods with lower fat content were more likely to report refreshing sleep (OR = 1.51, 95% CI: 1.49–1.52) compared to participants who did not choose these types of foods. However, it is important to note that the association between choosing foods with a lower fat content and refreshing sleep was not found to be statistically significant among participants without arthritis (*p*-value 0.708). Both participants with and without arthritis who reported choosing foods with a higher fiber content exhibited a higher likelihood of having refreshing sleep (participants with arthritis: OR = 1.15, 95% CI: 1.14–1.17; participants without arthritis: OR = 1.18, 95% CI: 1.18–1.19) compared to those who did not prioritize these foods. Additionally, participants who self-reported avoiding foods for cholesterol content were more likely to experience refreshing sleep (participants with arthritis: OR = 1.24, 95% CI: 1.23–1.26; participants without arthritis: OR = 1.19, 95% CI: 1.18–1.20). Interestingly, those who avoid foods for calorie content were more likely to report refreshing sleep (participants with arthritis: OR = 1.23, 95% CI:1.22–1.25; participants without arthritis: OR = 1.03, 95% CI: 1.03–1.04) compared to participants who did not avoid these foods (Table 4).

When examining the association between dietary choices and staying awake, our results find that participants who self-reported choosing foods with lower fat content were more likely to report difficulty staying awake (participants with arthritis: OR = 1.21, 95% CI:1.19–1.22; participants without arthritis: OR = 1.24, 95% CI: 1.24–1.25) compared to participants who did not. In the same way, the participants who self-reported choosing food with a higher fiber content were more likely to report difficulties staying awake (participants with arthritis: OR = 1.30, 95% CI: 1.28–1.31; participants without arthritis: OR = 1.25, 95% CI: 1.25–1.26) compared to participants who did not. The participants who self-reported avoiding foods for cholesterol content were more likely to have difficulties staying awake (participants with arthritis: OR = 1.26, 95% CI: 1.24–1.27; participants without arthritis: OR = 1.25, 95% CI: 1.24–1.26). Similarly, participants who were avoiding food for calorie content were more likely to report difficulties staying awake (participants with arthritis: OR = 1.14, 95% CI: 1.13–1.16; participants without arthritis: OR = 1.17, 95% CI: 1.16–1.17) compared to participants who were not avoiding such food (Table 4).

When assessing the association between dietary choices and trouble going to sleep, we found that participants with arthritis who self-reported choosing foods with a lower fat content were less likely to have trouble going to sleep (OR = 0.95, 95% CI: 0.94–0.96). However, participants without arthritis who self-reported choosing foods with a lower fat content were more likely to have trouble going to sleep (OR = 1.09, 95% CI: 1.08–1.09) compared to participants who did not. Furthermore, both participants with and without arthritis who self-reported choosing food for fiber content were more likely to report trouble going to sleep (participants with arthritis OR = 1.12, 95% CI: 1.11–1.14; participants without arthritis OR = 1.04, 95% CI: 1.04–1.05) compared to participants who did not. Additionally, participants who self-reported avoiding foods for cholesterol content were more likely to have trouble going to sleep (participants with arthritis: OR = 1.14, 95% CI: 1.13–1.16; participants without arthritis: OR = 1.13, 95% CI: 1.12–1.13). Similarly, those who were avoiding food for calorie content were more likely to report trouble going to sleep (participants with arthritis: OR = 1.07, 95% CI: 1.06–1.08; participants without arthritis: OR = 1.33, 95% CI: 1.32–1.33) compared to participants who were not avoiding such food (Table 4).

We also examined the association between sleep duration and self-reported dietary intake behaviors while adjusting for age, sex, BMI, and smoking and drinking status. Overall, participants without arthritis who self-reported choosing foods with lower fat content were less likely to sleep ≥7 h per night (OR = 0.96, 95% CI: 0.95–0.96) compared to participants who did not make such food choices. However, there was no statistically significant association for this relationship in participants with arthritis (*p*-value = 0.785). Regarding choosing food for fiber content, there was no statistically significant association observed between sleeping for ≥7 h per night and dietary choices based on fiber content among participants with arthritis (*p*-value = 0.064), but the participants without arthritis were more likely to sleep ≥7 h per night (OR = 1.06, 95% CI: 1.05–1.06) compared to participants who did not make such food choices. Regarding avoiding cholesterol-containing foods, participants with arthritis and those without arthritis who self-reported avoiding cholesterol-containing foods had lower odds of sleeping ≥7 h per night compared to participants who did not avoid such foods (participants with arthritis: OR = 0.88, 95% CI: 0.87–0.89; participants without arthritis: OR = 0.83, 95% CI: 0.83–0.84). Among participants without arthritis, those who self-reported avoiding foods for calorie content were 6% less likely to spend ≥7 h per night sleeping than those who did not avoid these foods (OR = 0.94, 95% CI: 0.93–0.94); however, this relationship was not significant in participants with arthritis (*p*-value = 0.301) (Table 4).

##### General Health and Dietary Intake Behaviors

When examining the association between self-reported general health and dietary intake behaviors, while adjusting for age, sex, BMI, and smoking and drinking status, the results indicated higher odds of perceiving general health as excellent/very good/good in both participants with and without arthritis who were choosing food with a lower fat content (with arthritis OR = 1.12, 95% CI: 1.10–1.13; without arthritis OR = 1.13, 95% CI: 1.12–1.14), choosing fiber content (with arthritis OR = 1.06, 95% CI:1.04–1.07; without arthritis OR = 1.11, 95% CI: 1.10–1.12), and avoiding calorie content (with arthritis OR = 1.20, 95% CI: 1.18–1.21; without arthritis OR = 1.38, 95% CI: 1.37–1.39) compared to those who did not choose those foods.. The participants without arthritis who were avoiding cholesterol containing foods were 9% less likely to perceive excellent/very good/good general health (OR = 0.91, 95% CI: 0.91–0.92); however, this association was not statistically significant in people with arthritis (*p*-value = 0.519) (Table 4).

##### Mental Health and Dietary Intake Behaviors

In investigating the associations between self-reported dietary intake behaviors and mental health (i.e., self-reported mental health, mood disorder, and anxiety disorder), adjustments were made for age, sex, BMI, and smoking and drinking status. The subsequent analysis revealed that participants with and without arthritis who were choosing food with a lower fat content were more likely to self-report excellent/very good/good mental health compared to participants who did not choose food with a lower fat content (with arthritis OR = 1.18, 95% CI: 1.16–1.20; without arthritis OR = 1.30, 95% CI: 1.29–1.31). The participants with arthritis who were choosing food with a higher fiber content were less likely to report that they perceived their mental health as excellent/very good/good (OR = 0.96, 95% CI: 0.95–0.98) compared to those who did not choose those foods, but the participants without arthritis who were choosing food with a higher fiber content were 54% more likely to self-report excellent/very good/good mental health (OR = 1.54, 95% CI: 1.52–1.55) compared to participants who did not choose those foods. The association between avoiding food for cholesterol content and excellent/very good/good mental health was not statistically significant among participants with arthritis (*p*-value: 0.063). However, the participants without arthritis who were avoiding food for cholesterol content were more likely to report that they perceived their mental health as excellent/very good/good (OR = 1.25, 95% CI: 1.24–1.26). Participants both with and without arthritis who were avoiding food for calorie content had higher odds of perceiving their mental health as excellent/very good/good (with arthritis OR = 1.28, 95% CI: 1.26–1.30; without arthritis OR = 1.38, 95% CI: 1.37–1.40) compared to those who did not avoid those foods.

When we examined the relation between self-reported dietary intake behaviors and mood disorder, our results found that the participants with arthritis who were choosing food with a lower fat content were more likely to report mood disorders (OR = 1.04, 95% CI: 1.02–1.05), but participants without arthritis were less likely to report mood disorders (OR = 0.88, 95% CI: 0.87–0.88) than participants who did not choose. Participants with arthritis who were choosing food with a higher fiber content were more likely to report that they suffered from a mood disorder (OR = 1.22, 95% CI: 1.20–1.24), but the participants without arthritis who were choosing food with a higher fiber content were less likely to have mood disorders (OR = 0.88, 95% CI: 0.88–0.89) compared to participants who did not make those food choices. The participants who were avoiding food for cholesterol content were less likely to experience mood disorders (with arthritis OR = 0.97, 95% CI: 0.95–0.98; without arthritis OR = 0.85, 95% CI: 0.84–0.86) compared to participants who were not. Furthermore, the participants who were avoiding food for calorie content were less likely to experience mood disorders (with arthritis OR = 0.91, 95% CI: 0.90–0.93; without arthritis OR = 0.83, 95% CI: 0.83–0.84) compared to participants who were not.

When we examined the association between self-reported dietary intake behaviors and anxiety disorders, our results showed that the participants with and without arthritis who were choosing food with a lower fat content were less likely to report anxiety disorders (with arthritis OR = 0.94, 95% CI: 0.93–0.96; without arthritis OR = 0.92, 95% CI: 0.91–0.93) compared to participants who were not. Participants with arthritis who were choosing food with a higher fiber content were more likely to report anxiety disorders (OR = 1.03, 95% CI: 1.01–1.05). However, the participants without arthritis who were choosing food with a higher fiber content were less likely to have anxiety disorders (OR = 0.94, 95% CI: 0.93–0.95). The participants with arthritis who were avoiding food for cholesterol content had higher odds of experiencing anxiety disorders (OR = 1.32, 95% CI: 1.30–1.34), but participants without arthritis who avoided food for cholesterol content were 8% less likely to report anxiety disorders (OR = 0.92, 95% CI: 0.91–0.93). Anxiety disorders were more likely to occur in participants with arthritis who avoided high-calorie-content foods (OR = 1.17, 95% CI: 1.15–1.19), but participants without arthritis who avoided high-calorie-content foods were 13% less likely to report that they had an anxiety disorder (OR = 0.87, 95% CI: 0.86–0.87) compared to participants who were not avoiding such foods (Table 4). It is important to highlight that that there was no overlapping population within the arthritis cohort that responded to both the dietary intake and oral health questions. Consequently, a comprehensive analysis in this context was not feasible.

#### 3.2.2. Self-Reported Health Status and Smoking

##### Sleep Quality and Smoking

When examining the association between sleep quality (refreshing sleep, difficulty staying awake, trouble going to sleep, and sleep duration) and smoking while adjusting for age, sex, BMI, and drinking status, the results showed that in comparison to never smokers, the participants with arthritis who were current smokers were less likely to have refreshing sleep (OR = 0.54, 95% CI: 0.53–0.54) and trouble going to sleep (OR = 0.99, 95% CI: 0.98–0.99), and more likely to report difficulties staying awake (OR = 1.14, 95% CI: 1.13–1.15) compared to non-smokers. The current smoker participants without arthritis were less likely to have refreshing sleep (OR = 0.63, 95% CI: 0.63–0.63) and more likely to report difficulty staying awake and trouble going to sleep (OR = 1.08, 95% CI: 1.08–1.08 and OR = 1.33, 95% CI: 1.32–1.33), respectively, compared to non-smokers. Current smokers were less likely to sleep ≥7 h per night (with arthritis OR = 0.91, 95% CI: 0.90–0.92; without arthritis OR = 0.80, 95% CI: 0.79–0.80), respectively, compared with non-smokers (Table 5).

Among participants with and without arthritis who were passive smokers, refreshing sleep was less likely to occur by 15% and 6%, respectively (with arthritis OR = 0.85, 95% CI: 0.83–0.86; without arthritis OR = 0.94, 95% CI: 0.93–0.95). Passive smokers with and without arthritis were both more likely to report difficulties staying awake (with arthritis OR = 1.12, 95% CI: 1.10–1.13; without arthritis OR = 1.07, 95% CI: 1.06–1.08) and more likely to have trouble going to sleep (with arthritis OR = 1.15, 95% CI: 1.13–1.17; without arthritis OR = 1.06, 95% CI: 1.05–1.06) compared to individuals who were not passive smokers (Table 5).

##### General Health and Smoking

When examining the association between general health and smoking among participants with and without arthritis, while adjusting for age, sex, BMI, and drinking status, the results found that participants with and without arthritis who were current smokers were less likely to self-report general health as excellent/very good/good (with arthritis OR = 0.41, 95% CI: 0.40–0.41; without arthritis OR = 0.35, 95% CI: 0.34–0.35) compared to non-smokers. Also, both groups of participants who smoked passively were less likely to self-report general health as excellent/very good/good (with arthritis OR = 0.69, 95% CI: 0.68–0.70; without arthritis OR = 0.69, 95% CI: 0.68–0.69) compared to individuals who were not passive smokers (Table 5).

##### Mental Health and Smoking

When examining the association between mental health and smoking while adjusting for age, sex, BMI, and drinking status, our results showed that individuals with and without arthritis who were current smokers were less likely to self-report excellent/very good/good mental health compared to individuals who never smoked (with arthritis OR = 0.41, 95% CI: 0.40–0.41; without arthritis OR = 0.40, 95% CI: 0.40–0.41). Both arthritis and non-arthritis groups who were current smokers were more likely to report mood disorders (with arthritis OR = 2.88, 95% CI: 2.86–2.89; without arthritis OR = 2.73, 95% CI: 2.72–2.74) and anxiety disorders (with arthritis OR = 2.87, 95% CI: 2.85–2.89; without arthritis OR = 2.62, 95% CI: 2.61–2.63) compared to non-smokers. Among passive smokers, participants with arthritis and those without arthritis were less likely to self-report mental health as excellent/very good/good by 36% and 24%, respectively (with arthritis OR = 0.64, 95% CI: 0.62–0.65; without arthritis OR = 0.76, 95% CI: 0.75–0.77) than those who did not passively smoke. Participants with and without arthritis who were passive smokers were more likely to have mood disorders (with arthritis OR = 1.18, 95% CI: 1.16–1.20; without arthritis OR = 1.54, 95% CI: 1.53–1.56) and anxiety disorders (with arthritis OR = 1.61, 95% CI: 1.58–1.64; without arthritis OR = 1.36, 95% CI: 1.35–1.38) compared to participants who were not passive smokers (Table 5).

##### Oral Health and Smoking

When examining the association between oral health and smoking among participants with and without arthritis, while adjusting for age, sex, BMI, and drinking status, the results showed that current smokers were less likely to have perceived oral health as excellent/very good/good (with arthritis OR = 0.36, 95% CI: 0.36–0.36; without arthritis OR = 0.27, 95% CI: 0.27–0.28).

Individuals who were current smokers were significantly more likely to have mouth pain (with arthritis OR = 1.47, 95% CI: 1.46–1.48; without arthritis OR = 1.81, 95% CI: 1.80–1.82), mouth dryness (with arthritis OR = 2.03, 95% CI: 2.02–2.04; without arthritis OR = 2.08, 95% CI: 2.07–2.08), and be uncomfortable while eating (with arthritis OR = 1.65, 95% CI: 1.63–1.66; without arthritis OR = 1.94, 95% CI: 1.93–1.95) compared to individuals who reported never smoking. However, participants with arthritis who were current and former smokers were less likely to have bleeding gums (OR = 0.71, 95% CI: 0.71–0.72 and OR = 0.97, 95% CI: 0.97–0.98, respectively). However, participants without arthritis who were current and former smokers were more likely to have bleeding gums (OR = 1.01, 95% CI: 1.00–1.01; OR = 1.03, 95% CI: 1.02–1.03, respectively) (Table 5).

Regarding passive smoking, respondents who reported this exposure were less likely to have perceived oral health as excellent/very good/good (with arthritis OR = 0.73, 95% CI: 0.71–0.74; without arthritis OR = 0.75, 95% CI: 0.74–0.76) compared to non-passive smokers. Also, these groups were significantly more likely to have mouth pain (with arthritis 84%, without arthritis 22%), bleeding gums (with arthritis 26%, without arthritis 3%), mouth dryness (with arthritis 46%, without arthritis 36%), and be uncomfortable while eating (with arthritis 27%, without arthritis 33%) in comparison to individuals who were not passive smokers (Table 5).

### 3.3. The Association between Sleep and Health Status in Participants with and without Arthritis

In examining the association between arthritis and refreshing sleep, our analysis revealed that individuals with arthritis were 32% less likely to report experiencing refreshing sleep (OR = 0.68, 95% CI 0.64–0.72) compared to those without arthritis (Table 3). When examining the association between refreshing sleep and general health, mental health, and oral health while adjusting for age, sex, BMI, and smoking and drinking status, our results showed that participants with and without arthritis who found their sleep refreshing were more likely to report good perceived general health (with arthritis OR = 2.96, 95% CI: 2.94–2.98; without arthritis OR = 3.57, 95% CI: 3.55–3.58), good perceived mental health (with arthritis OR = 4.32, 95% CI: 4.28–4.36; without arthritis OR = 3.76, 95% CI: 3.74–3.78), and good perceived oral health (with arthritis OR = 2.19, 95% CI: 2.16–2.22; without arthritis OR = 2.48, 95% CI: 2.46–2.50) compared to those who did not find their sleep refreshing (Table 6).

Furthermore, our results showed that people with arthritis were more likely to report difficulty staying awake (OR = 1.21, 95% CI 1.16–1.27) than people without arthritis (Table 3). When examining the association between difficulty staying awake and general health, mental health, and oral health while adjusting for age, sex, BMI, and smoking and drinking status, our results revealed that the people with and without arthritis who had difficulty staying awake were less likely to report good perceived general health (with arthritis OR = 0.53, 95% CI: 0.53–0.54; without arthritis OR = 0.55, 95% CI: 0.55–0.56), good perceived mental health (with arthritis OR = 0.41, 95% CI: 0.40–0.41; without arthritis OR = 0.47, 95% CI: 0.46–0.47), and good perceived oral health (with arthritis OR = 0.61, 95% CI: 0.60–0.62; without arthritis OR = 0.64, 95% CI: 0.64–0.65) compared to those who did not have difficulty staying awake (Table 6).

When exploring the association between arthritis and trouble going to sleep, our findings indicated that individuals with arthritis were 63% more likely to report trouble going to sleep (OR = 1.63, 95% CI 1.57–1.70) compared to those without arthritis (Table 3). Moreover, our results showed that the people with and without arthritis who had trouble going to sleep were less likely to report good perceived general health (with arthritis OR = 0.58, 95% CI: 0.58–0.59; without arthritis OR = 0.39, 95% CI: 0.38–0.39), good perceived mental health (with arthritis OR = 0.41, 95% CI: 0.41–0.42; without arthritis OR = 0.33, 95% CI: 0.33–0.33), and good perceived oral health (with arthritis OR = 0.72, 95% CI: 0.72–0.73; without arthritis OR = 0.54, 95% CI: 0.53–0.54) than those who did not have trouble going to sleep, while adjusting for age, sex, BMI, and smoking and drinking status (Table 6).

In examining the association between arthritis and sleep duration, our analysis revealed that people with arthritis were less likely to spend ≥7 h sleeping per night (OR = 0.79, 95% CI 0.76–0.82) than people without arthritis (Table 3). Additionally, in examining the association between sleep duration and general health, mental health, and oral health while adjusting for age, sex, BMI, and smoking and drinking status, our results showed that the participants with and without arthritis who spent ≥7 h sleeping per night were more likely to report good perceived general health (with arthritis OR = 1.59, 95% CI: 1.58–1.60; without arthritis OR = 1.37, 95% CI: 1.36–1.38), good perceived mental health (with arthritis OR = 1.60, 95% CI: 1.59–1.62; without arthritis OR = 1.54, 95% CI: 1.54–1.55), and good perceived oral health (with arthritis OR = 1.69, 95% CI: 1.67–1.71; without arthritis OR = 1.66, 95% CI: 1.65–1.67) than those spending <7 h per night sleeping (Table 6).

Supplementary Material S3 provides information about the associations between arthritis and sleep quality and duration for Models 1 and 2.

## 4. Discussion

The first objective of this study was to examine associations between arthritis and lifestyle traits, demographic characteristics, and self-reported health status. Additionally, we aim to explore associatios of lifestyle traits on the health-related conditions commonly associated with arthritis, including sleep quality, general health, mental health, and oral health in people with and without arthritis. This analysis considers previously studied individual-level covariates, such as age, sex, BMI, and alcohol intake to provide a comprehensive understanding of the complex relationships at play.

### 4.1. Arthritis, Demographic Characteristics, and Lifestyle Traits

The results of our study found statistically significant associations between arthritis and both sociodemographic characteristics (age, sex, education, marital status, and food security) and lifestyle traits (BMI, physical activity, smoking status, alcohol drinking status, and dietary behaviors) (Table 1 and Table 2). Consistent with previous studies, we found that women, individuals aged 65 years and older, and those with a low education level were more likely to self-report arthritis [1]. Additionally, people who were food insecure were more likely to self-report arthritis, which is in line with previous studies showing an association between food insecurity and health problems that cause functional limitation including arthritis [76,77,78]. This relationship may be due to limited access to healthcare, leading to poor disease management and exacerbation [78]. Also, functional disability (which can occur with arthritis) may affect the ability to work, resulting in less financial resources to purchase groceries, leading to food insecurity [78]. Our findings highlight an association between demographic characteristics and arthritis, emphasizing the need for targeted interventions and public health strategies to address these factors for arthritis management and prevention.

Our findings also showed a negative association between physical activity and arthritis. These findings are possibly due to lower activity levels among individuals who may be experiencing arthritis symptoms and/or who have concerns about exacerbating the condition. Nevertheless, health providers often encourage individuals with arthritis to be more engaged in physical activity [79], as research has shown that physical activity can have numerous benefits for people with arthritis including improved pain management, increased mobility, and enhanced overall quality of life [79,80]. According to the CDC, adults with arthritis should aim for 150 min per week of moderately intense activities or 75 min per week of vigorously intense aerobic activities [81]. This underscores the necessity for a public health policy aimed at guiding healthcare providers in effectively advising people with arthritis on the positive impact of physical activity for both disease management and overall well-being.

Our analysis also found a positive association between BMI and arthritis which is consistent with previous research [82,83]. This association is possibly because excess body weight places additional stress on the joints, which can lead to increased wear and tear over time. Moreover, adipose tissue produces cytokines and other inflammatory molecules that can contribute to the development of arthritis [84]. Thus, maintaining a healthy weight through regular physical activity and a balanced diet may reduce the risk of developing arthritis, and can also help manage symptoms in individuals who already have the condition.

In parallel with previous studies, our results demonstrated a negative association between alcohol consumption and arthritis; individuals with arthritis who consume alcohol tend to experience lower disease activity and a better quality of life [38,85]. Furthermore, Larsson et al. (2018) found that individuals with arthritis who stopped drinking had worse physical functionality and health-related quality of life, and more pain and fatigue compared to individuals who did not stop drinking alcohol [86]. However, relying on alcohol as a coping mechanism can lead to other long-term health problems including addiction, liver damage, cardiovascular issues, and mental health concerns [87]. Additionally, alcohol can interact with certain arthritis medications like Methotrexate, potentially reducing their effectiveness or causing adverse effects [88].

As in previous studies showing that smokers are at higher risk for arthritis, we found associations between both current and former smokers and arthritis compared to never smokers. We also found that this association was higher among former smokers compared to current smokers. There are a few possible explanations for why former smoking status may have a higher association with arthritis. Smoking cessation can lead to weight gain, and this extra weight can put additional stress on the joints, which may increase the association between arthritis and former smoking status [89]. On the other hand, in current smokers, the anti-inflammatory effect of nicotine may mask the symptoms of arthritis [90].

### 4.2. Health Status and Lifestyle Traits

#### 4.2.1. Sleep and Arthritis

In the CCHS, the quality of sleep is assessed through questions asking about the frequency of refreshing sleep, difficulty staying awake, and trouble going to sleep. The last two questions are used to assess insomnia symptoms in Canada through assessing daytime dysfunction and sleep onset latency, respectively [91,92]. Insomnia is defined as a complaint of difficulty going to sleep, trouble to maintaining sleep, or waking up too early, leading to non-restorative or poor-quality sleep [93]. Our analysis showed a negative association between arthritis and quality of sleep. We found that arthritis was negatively associated with refreshing sleep and an adequate sleep duration (≥7 h) and positively associated with difficulty staying awake and trouble going to sleep. These findings are in line with other studies showing a high prevalence of sleep disorders among individuals with rheumatoid arthritis and significant associations between disease activity and poor sleep quality [94,95]. The associations identified in our study underscore the importance of integrating sleep assessments and interventions into routine care for individuals with arthritis.

#### 4.2.2. Dietary Intake and Sleep in Arthritis

Our study showed that people with arthritis were more likely to self-report making healthier food choices including choosing food with a lower fat content and higher fiber content and avoiding food with a high cholesterol content compared to those without arthritis. This finding is in line with other studies showing a lower intake of discretionary foods in people with RA compared to the general population, which might be due to the fact that individuals with RA believe their food impacts their disease symptoms, and therefore they avoid these foods [51,96].

In this study, we hypothesized that the types of food chosen might not only affect arthritis but also have an association on other aspects of health status commonly associated with arthritis, such as sleep disturbance. To the best of our knowledge, this study is the first to assess the association between sleep quality and self-reported dietary behaviors in people with arthritis in Canada. In general, research suggests that there is a bidirectional association between sleep quality/quantity and dietary intake. Poor sleep quality can affect food choices and the desire to eat, and on the other hand, the type of nutrients we consume can influence sleep patterns in healthy people [97,98]. Our study found that choosing food with a lower fat content and avoiding cholesterol were associated with refreshing sleep in individuals with arthritis. This finding aligns with the results of a cross-sectional study that utilized data from a national survey in the United States which investigated the link between health, dietary intake, and sleep patterns. This study found that lower fat and cholesterol intake was correlated with better quality sleep and reduced daytime sleepiness in the general population [99]. Furthermore, consistent with our findings that indicated the association between a high fiber content and refreshing sleep, observational and interventional studies have found associations between a high fiber intake and deeper sleep (longer slow-wave sleep), which leads to better quality sleep [100,101]. Our study also found an association between avoiding food for calorie content and refreshing sleep in people with and without arthritis. However, some other studies have shown that caloric amounts, either high energy intake or caloric deprivation, have no significant impact on sleep quality or duration [102,103,104]. In general, studies on nutrition and sleep often exhibit high diversity and complexity in terms of participants and experimental conditions. These variations can contribute to conflicting study results, highlighting the need for more targeted and controlled investigations to draw definitive conclusions in these areas. Furthermore, sleep health is complex and comprises many dimensions, and not all dimensions are necessarily impacted by dietary intake at the same time.

#### 4.2.3. Mental Health and Arthritis

Our analysis found associations between self-reported mental health, anxiety, and mood disorder and arthritis. Studies have shown that sleep disturbance in people with arthritis is associated with both disease intensity and poor mental health issues, including anxiety and mood disorders [105,106,107]. Furthermore, chronic pain, regardless of the anatomical location, has an important relationship with anxiety and depression [108,109]. Therefore, it is not surprising that psychiatric disorders, including anxiety, mood disorders, and depression, occur with greater frequency among people with arthritis, as our study also found [21,108,109,110]. The important role of mental health for quality of life highlights the need to take measures to address or prevent mental health problems in this population. These findings emphasize the need for healthcare approaches that address both physical and mental well-being in individuals with arthritis.

#### 4.2.4. Mental Health and Dietary Intake in Arthritis

Over the last decade, we witnessed an exponential rise in the number of both prospective and cross-sectional studies in “nutritional psychiatry” documenting an association between diet quality and mental disorders [111,112]. Evidence supporting this association has also been steadily growing through discovering the important biological mediators affecting neurotransmission and enhancing neural signaling [113,114,115,116,117]. Our findings also found an association between mental health and self-reported dietary intake in individuals with and without arthritis. We found a positive association between choosing a low-fat diet and good general and mental health, as well as a negative association between choosing a low-fat diet and anxiety disorders. This finding is consistent with the study that indicated the long-term beneficial effect of a low-fat diet on mood state in participants with obesity and metabolic syndrome [118].

Cholesterol may have an impact on mental health due to its effect on neurotransmissions in the central nervous system [119]. Our study found a negative association between avoiding food for cholesterol content and mood disorders in people with and without arthritis which is consistent with studies showing a higher prevalence of depressive moods in individuals with higher serum total cholesterol levels [120,121]. Considering the dispute over the main contributory role of dietary cholesterol in elevated serum cholesterol and the complexities surrounding the relationship between dietary cholesterol and its impact on serum cholesterol [122], the association between serum cholesterol level and mood disorders cannot be concluded from our study.

Our study also revealed an association between choosing foods with high fiber and mental health issues, including self-reported mental health, anxiety, and mood disorders in individuals with arthritis. This aligns with existing research that documented the association of fiber intake or compliance to a high-fiber dietary pattern, like the Mediterranean Diet, with anxiety and depression [123,124,125,126]. It is proposed that dietary fiber has a beneficial effect on mental health through gut microbiota modulation that finally leads to a balanced gut microbiota–brain axis or a microbiota composition effect on neurotransmission [127,128,129]. However, it is noteworthy that a few other studies have reported inconsistent findings, indicating that the association may vary based on the study population or the source of dietary fiber [128,130].

Our analysis also found an association between avoiding food for calorie content and mental health, including self-reported mental health and mood disorders in individuals with and without arthritis. This finding is reinforced by evidence from both animal- and population-based studies underscoring the beneficial effects of a caloric restriction design on mood improvement [131,132]. One possible mechanism by which caloric restriction is believed to alleviate depressive syndrome is the activation of the hypothalamic–pituitary–adrenal axis and the increase in glucocorticoids, ultimately leading to depressive symptom alleviation [131,133].

#### 4.2.5. Smoking and Arthritis

Our study revealed a significant association between smoking (current or former) and arthritis in comparison to never smokers, which is consistent with prior research on smoking and various types of arthritis. Multiple studies have demonstrated the association between smoking and RA [134,135,136]. A meta-analysis study revealed that smokers are more likely to develop RA than non-smokers [27]. On the other hand, meta-analysis studies have indicated a negative association between smoking and osteoarthritis [137,138]. The variation in the association between smoking and arthritis may depend on the type of arthritis.

#### 4.2.6. Smoking/Passive Smoking and Sleep in Arthritis

Previous studies have shown that smokers were more likely to experience reduced sleep quality compared to non-smokers [54,139]. Our study found that smoking was associated with a poorer sleep quality among participants with and without arthritis. Specifically, both current and former smokers with arthritis were more likely to report poor sleep quality compared to never smokers. This association extends beyond active smoking to passive smoking, with numerous studies linking passive smoking to poorer sleep quality [140,141,142]. Our findings align with other studies showing that passive smokers with arthritis are more likely to report poor sleep quality compared to non-exposed individuals. It is well-established that sleep disturbance has been associated with different types of arthritis such as osteoarthritis (OA) [143] and RA [144]. Several studies have revealed an increase in pain, depression, and anxiety in people with RA following sleep disturbance [105,145]. In 2006, Luqmani et al. indicated that the early assessment and management of sleep disturbance is recommended to stabilize and monitor care for individuals with RA [146]. Given the association between sleep disturbance and smoking among participants with arthritis as shown in our study, addressing this link is crucial in developing effective treatment strategies for this group. However, further research is needed to better understand the underlying mechanisms and establish a robust causal relationship for targeted interventions.

#### 4.2.7. Smoking/Passive Smoking and Mental Health in Arthritis

The current study demonstrated a significant association between mental health and arthritis. Individuals with arthritis experienced a higher rate of mood and anxiety disorders than those without arthritis, consistent with previous research [147,148]. Moreover, several studies have shown a link between smoking and poor mental health [149,150,151]. In our investigation into the association of smoking and mental health in individuals with arthritis, current and former smokers with arthritis were more likely to have mood and anxiety disorders compared to non-smokers with arthritis. This association was less strong among former smokers with arthritis. These results are consistent with studies showing improved mental health following smoking cessation [152,153].

In addition, we found that passive smokers with arthritis were more likely to report mood and anxiety disorders than those not exposed to passive smoke. These results are in line with existing research that has demonstrated a higher risk of mood and anxiety disorders among smokers compared to non-smokers [149,154]. Canadian studies have specifically linked passive smoking to anxiety disorders, while other research has associated depression with passive smoking [155,156,157]. These findings underscore the importance of addressing smoking cessation and providing mental health support for arthritis management. Future studies should confirm the association between smoking and mental disorders among people with arthritis and investigate the underlying mechanisms that link smoking, mental health, and arthritis.

#### 4.2.8. Oral Health and Arthritis

In the current study, we found that participants with arthritis were 59% more likely to report poor oral health compared to participants without arthritis. These results are in accordance with previous studies indicating that people with RA have a poorer oral-health-related quality of life and have a higher risk of periodontal disease when compared to individuals without RA [158,159,160]. Also, various studies indicated that the risk of deteriorating oral health increased for people with RA and osteoarthritis [161,162].

#### 4.2.9. Smoking/Passive Smoking and Oral Health in Arthritis

Nearly every system and organ in the body is adversely affected by smoking, including the oral cavity. It is not surprising that smoking has the same adverse effect on oral health among people with arthritis. In 2007, Millar and Locker analyzed cycle 2.1 of the CCHS, and they showed that both current and former smokers were more likely to have oral health problems than those who had never smoked [163]. Many studies have indicated that smoking is associated with poor oral health, including the prevalence and severity of periodontal diseases [164,165,166]. Our study confirmed that current and former smokers with arthritis were more likely to exhibit poor oral health than non-smokers with arthritis. Additionally, individuals with arthritis who had been exposed to passive smoking were more likely to report poor oral health compared to those with arthritis who had not been exposed to passive smoking. Our study’s findings align with studies that demonstrated an association between passive smoking and oral health issues including oral cancer [167,168,169]. Interestingly, despite the detrimental effect of smoking on oral health, our study showed that participants with arthritis who smoked were less likely to experience bleeding gums than non-smokers. Also, former smokers with arthritis were less likely to report bleeding gums than non smoker, aligning with research indicating smoking can mask signs of periodontitis and reduces gum bleeding [170]. Also, the study showed that smoking cessation increases gum bleeding [171].

Arthritis negatively affects oral health, contributing to dental caries and gingivitis, and temporomandibular joint problems, often resulting in mouth pain in individuals with arthritis [172,173,174]. Moreover, it has been well established that smoking increases the risk of mouth pain [163,175]. To our knowledge, no study has been conducted that reports the association between smoking and mouth pain among people with arthritis. Our study revealed that people with arthritis who were current or former smokers as well as passive smokers suffered from mouth pain more than people with arthritis who had never smoked.

Our study revealed a significant association between arthritis and dry mouth. Specifically, participants with arthritis were 170% more likely to report dry mouth compared to individuals without arthritis. This aligns with studies linking autoimmune diseases to salivary gland dysfunction and xerostomia [176,177,178]. Moreover, our current study showed that current and former smokers with arthritis were more likely to report dry mouth than non-smokers with arthritis, consistent with previous research linking dry mouth to smoking [179,180]. A previous study indicated that individuals suffering from dry mouth experience oral symptoms such as dental caries, gingivitis, and mastication problems [181], which were more pronounced among smoker participants with arthritis in our study.

The association between smoking and oral health problems among people with arthritis that was shown in this study underscores the importance for dentists to create proper treatment plans for these individuals. Future studies should focus on investigate underlying mechanisms and evaluate smoking cessation interventions. These studies can enhance our understanding and inform targeted interventions to improve oral health in individuals with arthritis.

### 4.3. Sleep and Health Status

The current study underscores the association between arthritis and poor sleep quality and duration. Moreover, in examining the association of sleep quality and duration with perceived general health, mental health, and oral health among people with arthritis, we found that people with arthritis who reported experiencing refreshing sleep were significantly more likely to report positive perceptions across all health statuses than those who did not find their sleep refreshing. Conversely, people with arthritis facing difficulties staying awake or trouble going to sleep were less likely to report positive perceived general health, mental health, and oral health than those who did not face these issues. Additionally, an adequate sleep duration (≥7 h per night) was associated with positive perceived health status compared to an inadequate sleep duration (<7 h per night). The identified associations between sleep and health status elucidated in our study underscores the importance of adopting comprehensive and personalized approaches in the prevention and care of arthritis. The circadian clock plays a crucial role in the regulation of various physiological process, including those that can be affected by arthritis [182]. There is a consensus that the disruption of circadian rhythms can have a profound effect on human health status and well-being [183,184,185]. Epidemiological studies have shown that individuals who work night/irregular shifts or have irregular sleep patterns are at increased risk for metabolic disorders, such as obesity and type 2 diabetes, cardiovascular disease, cancer, and neurodegenerative and autoimmune conditions [186,187,188]. Emerging studies have suggested a key role of the circadian clock in the onset and disease progression of arthritis, along with nascent evidence suggesting that optimizing individual circadian rhythms through lifestyle interventions may benefit those patients. This study can contribute to an increased understanding on the links between circadian disruption, susceptibility to disease, and disease development and progression in support of potential therapeutic opportunities for a range of medical conditions including arthritis, as well as strategies for preventing circadian-related health conditions and improving clinical outcomes through lifestyle changes.

### 4.4. Strengths and Limitations

A significant strength of this study lies in its robust study design, which meticulously encompasses various variables, effectively addressing two pivotal research aims within the framework of one distinctive population study. Moreover, this study is the first of its kind, not only in examining associations between lifestyle traits and arthritis, but also their association with health status commonly associated with arthritis among the Canadian population. This research utilizes a large, reliable survey, contributing to the existing body of knowledge by providing novel insights into the intricate associations between lifestyle traits and various types of health status measures in individuals with arthritis.

The primary limitation of this study is its cross-sectional design, limiting the capacity to determine a temporal link or cause-and-effect relationship between outcome and exposure. One constraint of this survey stems from a lack of detailed information about the specific type and stage of arthritis, the duration of the disease, and the treatments used. Because of this lack of information, there is likely a wide variation in disease characteristics among respondents who have confirmed arthritis. As a result, the survey’s reliability and the clarity of the results are affected by this significant diversity among individuals who responded positively to the arthritis-related question. Another limitation of our study the lack of participants who completed both questions on oral health status and dietary intake. This lack of data prevents a comprehensive analysis of these important associations. This study also relies on self-reported data from the CCHS, which can introduce biases and inaccuracies. The absence of objective health indicators is a limitation. Future research should include objective health markers alongside self-reported data for a more comprehensive understanding. Furthermore, one challenge for surveys using self-reported questionnaires is answer reliability, as participants may make more socially acceptable choices rather than providing the true answers. In addition, the questions asking about self-reported dietary intake only ask participants to report information about self-reported choices of foods based on single dietary components and, and therefore do not provide a well-recognized, valid estimation of intake. Furthermore, we expect that some respondents may have had difficulties answering some of these questions about food choices as many members of the public have limited awareness of food sources of different nutrients. In addition, the examination of the association between a single macronutrient and health status may generate inconsistent results, as often the intake of one macronutrient leads to changes in other macronutrients. These limitations highlight the need for a cautious interpretation of our results and emphasize the importance of addressing methodological limitations in future research on dietary influences.

## 5. Conclusions

This study represents an in-depth exploration of possible associations between lifestyle traits and health status in Canadian participants with arthritis. Employing a meticulous analysis, we have unveiled noteworthy associations that carry substantial potential implications for both the prevention and management of arthritis.

Our study brought to light the associations between arthritis and demographic characteristics, revealing the heightened susceptibility of women, older individuals, and those with lower educational levels. The investigation extended to health statuses commonly associated with arthritis, uncovering the association between arthritis and poor sleep quality and poor general health, mental health, and oral health. Furthermore, our study explored associations between dietary choices and general health, mental health, and sleep quality in individuals with arthritis. Additionally, our findings highlight the association between smoking (active and passive) and poor sleep quality and poor general health, mental health, and oral health in people with arthritis.

## Figures and Tables

**Table 1:** Sociodemographic characteristics of participants with and without arthritis.

Variable (*N* = 104,359)	W/O Arthritis (%) 76,773 (73.6%)	With Arthritis (%) 27,586 (26.4%)	OR ^†^ (95% CI)	*p*-Value
Sex, *n* = 104,359 (100% out of *N*)				
Male	37,226 (48.5%)	10,620 (38.5%)	1.00 (ref)	
Female	39,547 (51.5%)	16,966 (61.5%)	1.50 (1.46–1.55)	<0.001
Age (years), *n* = 104,359 (100% out of *N*)				
18–34	21,427 (27.9%)	659 (2.4%)	1.00 (ref)	
35–49	19,427 (25.3%)	2449 (8.9%)	4.10 (3.75–4.48)	<0.001
50–64	19,419 (25.3%)	8700 (31.5%)	14.57 (13.43–15.80)	<0.001
≥65	16,500 (21.5%)	15,778 (57.2%)	31.09 (28.69–33.70)	<0.001
Marital status, *n* = 104,118 (99.8% out of *N*)				
Single	20,381 (26.6%)	3460 (12.6%)	1.00 (ref)	
Married/common law	43,011 (56.2%)	14,265 (51.8%)	1.95 (1.88–2.04)	<0.001
Widowed/divorced/separated	13,210 (17.2%)	9791 (35.6%)	4.37 (4.18–4.57)	<0.001
Education, *n* = 102,832 (98.5% out of *N*)				
Post-secondary, diploma, or university degree	48,134 (63.5%)	14,129 (52.2%)	1.00 (ref)	
Secondary school graduation	18,309 (24.2%)	6299 (23.3%)	1.17 (1.13–1.21)	<0.001
Less than secondary school graduation	9327 (12.3%)	6634 (24.5%)	2.42 (2.34–2.51)	<0.001
Food Security, *n* = 102,848 (98.6% out of *N*)				
Food secure	69,251 (91.5%)	24,437 (90.1%)	1.00 (ref)	
Moderately/severely food insecure	6470 (8.5%)	2690 (9.9%)	1.18 (1.12–1.24)	<0.001

Sociodemographic characteristics of participants with and without arthritis of the Canadian cohort aged 18 years and older (Canadian Community Health Survey, 2017–2018). Data presented as odds ratios and 95% confidence intervals. † Logistic regression. Data considered statistically significant when *p*-value ≤ 0.05. *N* = total participants; *n* = participants within variable; W/O = without; OR = odds ratio; CI = confidence interval; ref = reference.

**Table 2:** Lifestyle traits of participants with and without arthritis.

Variable	W/O Arthritis (%)	With Arthritis (%)	OR ^†^ (95% CI)	*p*-Value
Self-reported BMI, *n* = 97,768 (93.7% out of *N*)				
Normal weight	30,784 (42.6%)	7821 (30.7%)	1.00 (ref)	
Underweight	1480 (2.1%)	410 (1.6%)	1.09 (0.98–1.22)	0.130
Overweight	25,028 (34.6%)	9241 (36.2%)	1.45 (1.40–1.50)	<0.001
Obese—Class I, II, III	14,974 (20.7%)	8030 (31.5%)	2.11 (2.04–2.19)	<0.001
Physical activity indicator, *n* = 102,350 (98.1% out of *N*)				
≥CPAG level	42,713 (56.6%)	11,466 (42.7%)	1.00 (ref)	
<CPAG level	17,384 (23.0%)	6265 (23.3%)	1.34 (1.30–1.39)	<0.001
No physical activity	15,373 (20.4%)	9149 (34.0%)	2.22 (2.15–2.29)	<0.001
Alcohol Drinking, *n* = 103,926 (99.6% out of *N*)				
No drinking in the last year	13,869 (18.1%)	7542 (27.5%)	1.00 (ref)	
Occasional	12,643 (16.6%)	5209 (19.0%)	0.76 (0.73–0.79)	<0.001
Regular	49,959 (65.3%)	14,704 (53.5%)	0.54 (0.52–0.56)	<0.001
Food Choice—Choosing lower fat content, *n* = 13,740 (13.1% of *N*)				
Not choosing	4376 (42.3%)	1175 (34.6%)	1.00 (ref)	
Choosing	5969 (57.7%)	2220 (65.4%)	1.39 (1.28–1.50)	<0.001
Food Choice—Choosing fiber content, *n* = 13,737 (13.1% of *N*)				
Not choosing	4186 (40.5%)	1075 (31.6%)	1.00 (ref)	
Choosing	6152 (59.5%)	2324 (68.4%)	1.47 (1.36–1.60)	<0.001
Food Choice—Avoiding cholesterol content, *n* = 13,714 (13.1% of *N*)				
Not avoiding	6148 (59.5%)	1802 (53.2%)	1.00 (ref)	
Avoiding	4177 (40.5%)	1587 (46.8%)	1.30 (1.20–1.40)	<0.001
Food Choice—Avoiding calorie content, *n* = 13,734 (13.1% of *N*)				
Not avoiding	4962 (48.0%)	1595 (47.0%)	1.00 (ref)	
Avoiding	5378 (52.0%)	1799 (53.0%)	1.04 (0.96–1.13)	0.315
Total daily consumption of fruits and vegetables, *n* = 2241 (2.1% out of *N*)				
≥5 times	442 (24.4%)	101 (23.7%)	1.00 (ref)	
<5 times	1373 (75.6%)	325 (76.3%)	1.04 (0.81–1.33)	0.780
Smoking, *n* = 103,935 (99.6% out of *N*)				
Never	40,974 (53.6%)	11,328 (41.3%)	1.00 (ref)	
Current	14,638 (19.1%)	4852 (17.7%)	1.20 (1.15–1.25)	<0.001
Former	20,914 (27.3%)	11,229 (41.0%)	1.94 (1.88–2.00)	<0.001
Exposure to second hand smoke, *n* = 27,528 (26.3% of *N*)				
Not passive smoker	18,691 (90.8%)	6155 (88.5%)	1.00 (ref)	
Passive smoker	1885 (9.2%)	797 (11.5%)	1.28 (1.18–1.40)	<0.001

Lifestyle traits of participants with and without arthritis of the Canadian cohort aged 18 years and older (Canadian Community Health Survey, 2017–2018). Data presented as odds ratios and 95% confidence intervals. † Logistic regression. Data considered statistically significant when *p*-value ≤ 0.05. CPAG = Canada’s Physical Activity Guide; BMI = body mass index; *N* = total participants; *n* = participants within variable; W/O = without; OR = odds ratio; CI = confidence interval; ref = reference.

**Table 3:** Health status of participants with and without arthritis.

Variable			W/O Arthritis (%)	With Arthritis (%)	OR ^†^ (95% CI)	*p*-Value
Sleep Quality						
Refreshing sleep *n* = 50,918 (48.8% out of *N*)		No	5311 (13.7%)	2336 (19.0%)	1.00 (ref)	
		Yes	33,327 (86.3%)	9944 (81.0%)	0.68 (0.64–0.72)	<0.001
Difficulty staying awake *n* = 50,849 (48.7% out of *N*)		No	28,346 (73.5%)	8528 (69.6%)	1.00 (ref)	
		Yes	10,245 (26.5%)	3730 (30.4%)	1.21 (1.16–1.27)	<0.001
Having trouble going to sleep *n* = 51,018 (48.9% out of *N*)		No	20,454 (52.8%)	5018 (40.7%)	1.00 (ref)	
		Yes	18,248 (47.2%)	7298 (59.3%)	1.63 (1.57–1.70)	<0.001
Spent ≥7 h per night sleeping *n* = 50,834 (48.7% out of *N*)		No	15,801 (40.9%)	5734 (46.8%)	1.00 (ref)	
		Yes	22,788 (59.1%)	6511 (53.2%)	0.79 (0.76–0.82)	<0.001
General Health Condition						
General health *n* = 104,198 (99.8% out of *N*)		Good	69,377 (90.6%)	19,584 (71.2%)	1.00 (ref)	
		Poor	7303 (9.5%)	7934 (28.8%)	3.85 (3.71–3.99)	<0.001
Diabetes *n* = 104,254 (99.9% out of *N*)		No	71,314 (93.0%)	22,923 (83.2%)	1.00 (ref)	
		Yes	5393 (7.0%)	4624 (16.8%)	2.67 (2.56–2.78)	<0.001
Cancer *n* = 104,177 (99.8% out of *N*)		No	75,375 (98.3%)	26,450 (96.2%)	1.00 (ref)	
		Yes	1304 (1.7%)	1048 (3.8%)	2.29 (2.11–2.49)	<0.001
High total cholesterol *n* = 102,983 (98.7% out of *N*)		No	67,143 (88.4%)	19,890 (23.5%)	1.00 (ref)	
		Yes	8793 (11.6%)	7157 (26.5%)	2.75 (2.65–2.85)	<0.001
High blood pressure *n* = 104,009 (99.7% out of *N*)		No	63,297 (82.7%)	16,294 (59.3%)	1.00 (ref)	
		Yes	13,237 (17.3%)	11,181 (40.7%)	3.28 (3.18–3.38)	<0.001
Mental Health Condition						
Mental health *n* = 101,540 (97.3% out of *N*)		Good	69,782 (93.0%)	23,618 (89.2%)	1.00 (ref)	
		Poor	5272 (7.0%)	2868 (10.8%)	1.61 (1.53–1.69)	<0.001
Mood disorder *n* = 104,170 (99.8% out of *N*)		No	69,915 (91.2%)	23,669 (86.0%)	1.00 (ref)	
		Yes	6730 (8.8%)	3856 (14.0%)	1.69 (1.62–1.77)	<0.001
Anxiety disorder *n* = 104,148 (99.8% out of *N*)		No	69,963 (91.3%)	24,151 (87.8%)	1.00 (ref)	
		Yes	6674 (8.7%)	3360 (12.2%)	1.46 (1.40–1.52)	<0.001
Oral Health Condition						
Oral health *n* = 52,510 (50.3% out of *N*)		Good	35,190 (90.3%)	11,580 (85.5%)	1.00 (ref)	
		Poor	3769 (9.7%)	1971 (14.5%)	1.59 (1.50–1.69)	<0.001
Bleeding gums *n* = 47,468 (45.5% out of *N*)		No	26,893 (74.0%)	8947 (80.4%)	1.00 (ref)	
		Yes	9445 (26.0%)	2183 (19.6%)	0.70 (0.66–0.73)	<0.001
Mouth pain *n* = 52,520 (50.1% out of *N*)		No	34,837 (89.4%)	11,554 (85.2%)	1.00 (ref)	
		Yes	4126 (10.6%)	2003 (14.8%)	1.46 (1.38–1.55)	<0.001
Mouth dryness *n* = 52,463 (50.3% out of *N*)		No	31,876 (81.9%)	8491 (62.7%)	1.00 (ref)	
		Yes	7041 (18.1%)	5055 (37.3%)	2.70 (2.58–2.82)	<0.001
Uncomfortable eating *n* = 52,515 (50.3% out of *N*)		No	33,025 (84.8%)	10,649 (78.6%)	1.00 (ref)	
		Yes	5938 (15.2%)	2903 (21.4%)	1.52 (1.44–1.59)	<0.001

Health condition of participants with and without arthritis of the Canadian cohort (Canadian Community Health Survey, 2017–2018). Data presented as odds ratios (ORs) and 95% confidence intervals. † Logistic regression. Data considered statistically significant when *p*-value ≤ 0.05. *N* = total participants; *n* = participants within variable; W/O = without; OR = odds ratio; CI = confidence interval; ref = reference.

**Table 4:** Association between sleep quality, general health and mental health with dietary intake in participants with and without arthritis.

Variables	Population	OR (95% CI)	*p*-Value
Refreshing sleep			
Individuals who choose lower fat content (ref: not choosing)	Arthritis (*n* = 3227)	1.51 (1.49–1.52)	<0.001
	W/O arthritis (*n* = 9900)	1.00 (0.99–1.01)	0.708
Individuals who choose fiber content (ref: not choosing)	Arthritis (*n* = 3230)	1.15 (1.14–1.17)	<0.001
	W/O arthritis (*n* = 9892)	1.18 (1.18–1.19)	<0.001
Individuals who avoid cholesterol content (ref: not avoiding)	Arthritis (*n* = 3219)	1.24 (1.23–1.26)	<0.001
	W/O arthritis (*n* = 9882)	1.19 (1.18–1.20)	<0.001
Individuals who avoid calorie content (ref: not avoiding)	Arthritis (*n* = 3227)	1.23 (1.22–1.25)	<0.001
	W/O arthritis (*n* = 9896)	1.03 (1.03–1.04)	<0.001
Difficulty staying awake			
Individuals who choose lower fat content (ref: not choosing)	Arthritis (*n* = 3215)	1.21 (1.19–1.22)	<0.001
	W/O arthritis (*n* = 9876)	1.24 (1.24–1.25)	<0.001
Individuals who choose fiber content (ref: not choosing)	Arthritis (*n* = 3217)	1.30 (1.28–1.31)	<0.001
	W/O arthritis (*n* = 9868)	1.25 (1.25–1.26)	<0.001
Individuals who avoid cholesterol content (ref: not choosing)	Arthritis (*n* = 3207)	1.26 (1.24 –1.27)	<0.001
	W/O arthritis (*n* = 9859)	1.25 (1.24–1.26)	<0.001
Individuals who avoid calorie content (ref: not avoiding)	Arthritis (*n* = 3214)	1.14 (1.13–1.16)	<0.001
	W/O arthritis (*n* = 9873)	1.17 (1.16–1.17)	<0.001
Trouble going to sleep			
Individuals who choose lower fat content (ref: not choosing)	Arthritis (*n* = 3228)	0.95 (0.94–0.96)	<0.001
	W/O arthritis (*n* = 9908)	1.09 (1.08–1.09)	<0.001
Individuals who choose fiber content (ref: not choosing)	Arthritis (*n* = 3231)	1.12 (1.11–1.14)	<0.001
	W/O arthritis (*n* = 9900)	1.04 (1.04–1.05)	<0.001
Individuals who avoid cholesterol content (ref: not avoiding)	Arthritis (*n* = 3221)	1.14 (1.13–1.16)	<0.001
	W/O arthritis (*n* = 9890)	1.13 (1.12–1.13)	<0.001
Individuals who avoid calorie content (ref: not avoiding)	Arthritis (*n* = 3228)	1.07 (1.06–1.08)	<0.001
	W/O arthritis (*n* = 9904)	1.33 (1.32–1.33)	<0.001
Spent ≥7 h per night sleeping			
Individuals who choose lower fat content (ref: not choosing)	Arthritis (*n* = 3204)	1.00 (0.99–1.01)	0.785
	W/O arthritis (*n* = 9878)	0.96 (0.95–0.96)	<0.001
Individuals who choose fiber content (ref: not choosing)	Arthritis (*n* = 3206)	1.01 (1.00–1.02)	0.064
	W/O arthritis (*n* = 9870)	1.06 (1.05–1.06)	<0.001
Individuals who avoid cholesterol content (ref: not avoiding)	Arthritis (*n* = 3196)	0.88 (0.87–0.89)	<0.001
	W/O arthritis (*n* = 9860)	0.83 (0.83–0.84)	<0.001
Individuals who avoid calorie content (ref: not avoiding)	Arthritis (*n* = 3203)	1.00 (0.98–1.01)	0.301
	W/O arthritis (*n* = 9874)	0.94 (0.93–0.94)	<0.001
Perceived general health			
Individuals who choose lower fat content (ref: not choosing)	Arthritis (*n* = 3236)	1.12 (1.10–1.13)	<0.001
	W/O arthritis (*n* = 9908)	1.13 (1.12–1.14)	<0.001
Individuals who choose fiber content (ref: not choosing)	Arthritis (*n* = 3240)	1.06 (1.04–1.07)	<0.001
	W/O arthritis (*n* = 9901)	1.11 (1.10–1.12)	<0.001
Individuals who avoid cholesterol content (ref: not avoiding)	Arthritis (*n* = 3229)	1.00 (0.99–1.02)	0.519
	W/O arthritis (*n* = 9889)	0.91 (0.91–0.92)	<0.001
Individuals who avoid calorie content (ref: not avoiding)	Arthritis (*n* = 3235)	1.20 (1.18–1.21)	<0.001
	W/O arthritis (*n* = 9903)	1.38 (1.37–1.39)	<0.001
Perceived mental health			
Individuals who choose lower fat content (ref: not choosing)	Arthritis (*n* = 3228)	1.18 (1.16–1.20)	<0.001
	W/O arthritis (*n* = 9905)	1.30 (1.29–1.31)	<0.001
Individuals who choose fiber content (ref: not choosing)	Arthritis (*n* = 3231)	0.96 (0.95–0.98)	<0.001
	W/O arthritis (*n* = 9896)	1.54 (1.52–1.55)	<0.001
Individuals who avoid cholesterol content (ref: not avoiding)	Arthritis (*n* = 3221)	0.99 (0.97–1.00)	0.063
	W/O arthritis (*n* = 9885)	1.25 (1.24–1.26)	<0.001
Individuals who avoid calorie content (ref: not avoiding)	Arthritis (*n* = 3227)	1.28 (1.26–1.30)	<0.001
	W/O arthritis (*n* = 9900)	1.38 (1.37–1.40)	<0.001
Mood disorder			
Individuals who choose lower fat content (ref: not choosing)	Arthritis (*n* = 3233)	1.04 (1.02–1.05)	<0.001
	W/O arthritis (*n* = 9904)	0.88 (0.87–0.88)	<0.001
Individuals who choose fiber content (ref: not choosing)	Arthritis (*n* = 3236)	1.22 (1.20–1.24)	<0.001
	W/O arthritis (*n* = 9896)	0.88 (0.88–0.89)	<0.001
Individuals who avoid cholesterol content (ref: not avoiding)	Arthritis (*n* = 3226)	0.97 (0.95–0.98)	<0.001
	W/O arthritis (*n* = 9886)	0.85 (0.84–0.86)	<0.001
Individuals who avoid calorie content (ref: not avoiding)	Arthritis (*n* = 3232)	0.91 (0.90–0.93)	<0.001
	W/O arthritis (*n* = 9899)	0.83 (0.83–0.84)	<0.001
Anxiety disorder			
Individuals who choose lower fat content (ref: not choosing)	Arthritis (*n* = 3231)	0.94 (0.93–0.96)	<0.001
	W/O arthritis (*n* = 9904)	0.92 (0.91–0.93)	<0.001
Individuals who choose fiber content (ref: not choosing)	Arthritis (*n* = 3234)	1.03 (1.01–1.05)	<0.001
	W/O arthritis (*n* = 9896)	0.94 (0.93–0.95)	<0.001
Individuals who avoid cholesterol content (ref: not avoiding)	Arthritis (*n* = 3224)	1.32 (1.30–1.34)	<0.001
	W/O arthritis (*n* = 9886)	0.92 (0.91–0.93)	<0.001
Individuals who avoid calorie content (ref: not avoiding)	Arthritis (*n* = 3230)	1.17 (1.15–1.19)	<0.001
	W/O arthritis (*n* = 9899)	0.87 (0.86–0.87)	<0.001

Association of sleep quality, general health, and mental health with dietary intake in participants with and without arthritis. Binary logistic regression adjusted for age, sex, BMI, smoking status, and drinking status (Model 3). Data presented as odds ratios (ORs) and 95% confidence intervals; all analyses were weighted. Data considered statistically significant when *p*-value ≤ 0.05. W/O = without; OR = odds ratio; CI = confidence interval; *n* = number of respondents; ref = reference.

**Table 5:** Association between sleep quality, general health, mental health, and oral health with smoking/passive smoking in participants with and without arthritis.

Variables	Population		OR (95% CI)	*p*–Value
Smokers (ref: never smoker)				
Refreshing sleep in individuals who are a current or former smoker	Arthritis *n* = 11,818	Current	0.54 (0.53–0.54)	<0.001
		Former	0.79 (0.78–0.80)	<0.001
	W/O arthritis *n* = 37,124	Current	0.63 (0.63–0.63)	<0.001
		Former	0.85 (0.84–0.85)	<0.001
Difficulty staying awake in individuals who are a current or former smoker	Arthritis *n* = 11,798	Current	1.14 (1.13–1.15)	<0.001
		Former	0.94 (0.93–0.94)	<0.001
	W/O arthritis *n* = 37,097	Current	1.08 (1.08–1.08)	<0.001
		Former	0.92 (0.92–0.92)	<0.001
Trouble going to sleep in individuals who are a current or former smoker	Arthritis *n* = 11,851	Current	0.99 (0.98–0.99)	<0.001
		Former	1.13 (1.12–1.14)	<0.001
	W/O arthritis *n* = 37,184	Current	1.33 (1.32–1.33)	<0.001
		Former	1.18 (1.17–1.18)	<0.001
Spent ≥7 h per night sleeping in individuals who are a current or former smoker	Arthritis *n* = 11,790	Current	0.91 (0.90–0.92)	<0.001
		Former	1.05 (1.04–1.05)	<0.001
	W/O arthritis *n* = 37,104	Current	0.80 (0.79–0.80)	<0.001
		Former	0.97 (0.97–0.98)	<0.001
Perceived General health in individuals who are a current or former smoker	Arthritis *n* = 25,223	Current	0.41 (0.40–0.41)	<0.001
		Former	0.76 (0.76–0.77)	<0.001
	W/O arthritis *n* = 71,814	Current	0.35 (0.34–0.35)	<0.001
		Former	0.68 (0.68–0.68)	<0.001
Perceived mental health in individuals who are a current or former smoker	Arthritis *n* = 25,209	Current	0.41 (0.40–0.41)	<0.001
		Former	0.84 (0.83–0.84)	<0.001
	W/O arthritis *n* = 71,801	Current	0.40 (0.40–0.41)	<0.001
		Former	0.84 (0.84–0.84)	<0.001
Mood disorder in individuals who are a current or former smoker	Arthritis *n* = 25,236	Current	2.88 (2.86–2.89)	<0.001
		Former	1.43 (1.42–1.44)	<0.001
	W/O arthritis *n* = 71,798	Current	2.73 (2.72–2.74)	<0.001
		Former	1.58 (1.57–1.59)	<0.001
Anxiety disorder in individuals who are a current or former smoker	Arthritis *n* = 25,221	Current	2.87 (2.85–2.89)	<0.001
		Former	1.30 (1.29–1.30)	<0.001
	W/O arthritis *n* = 71,801	Current	2.62 (2.61–2.63)	<0.001
		Former	1.63 (1.63–1.64)	<0.001
Perceived oral health in individuals who are a current or former smoker	Arthritis *n* = 12,908	Current	0.36 (0.36–0.36)	<0.001
		Former	0.93 (0.92–0.94)	<0.001
	W/O arthritis *n* = 37,275	Current	0.27 (0.27–0.28)	<0.001
		Former	0.68 (0.68–0.68)	<0.001
Mouth pain in individuals who are a current or former smoker	Arthritis *n* = 12,914	Current	1.47 (1.46–1.48)	<0.001
		Former	1.11 (1.10–1.11)	<0.001
	W/O arthritis *n* = 37,275	Current	1.81 (1.80–1.82)	<0.001
		Former	1.22 (1.22–1.23)	<0.001
Bleeding gums in individuals who are a current or former smoker	Arthritis *n* = 10,597	Current	0.71 (0.71–0.72)	<0.001
		Former	0.97 (0.97–0.98)	<0.001
	W/O arthritis *n* = 34,750	Current	1.01 (1.00–1.01)	0.009
		Former	1.03 (1.02–1.03)	<0.001
Mouth dryness in individuals who are a current or former smoker	Arthritis *n* = 12,905	Current	2.03 (2.02–2.04)	<0.001
		Former	1.24 (1.23–1.25)	<0.001
	W/O arthritis *n* = 37,235	Current	2.08 (2.07–2.08)	<0.001
		Former	1.39 (1.39–1.40)	<0.001
Uncomfortable eating in individuals who are a current or former smoker	Arthritis *n* = 12,908	Current	1.65 (1.63–1.66)	<0.001
		Former	1.15 (1.14–1.15)	<0.001
	W/O arthritis *n* = 37,274	Current	1.94 (1.93–1.95)	<0.001
		Former	1.39 (1.39–1.40)	<0.001
Passive smokers (ref: not passive smoker)				
Refreshing sleep in individuals who report passive smoke exposure	Arthritis (*n* = 5533)		0.85 (0.83–0.86)	<0.001
	W/O arthritis (*n* = 17,327)		0.94 (0.93–0.95)	<0.001
Difficulty staying awake in individuals who report passive smoke exposure	Arthritis (*n* = 5524)		1.12 (1.10–1.13)	<0.001
	W/O arthritis (*n* = 17,327)		1.07 (1.06–1.08)	<0.001
Trouble going to sleep in individuals who report passive smoke exposure	Arthritis (*n* = 5551)		1.15 (1.13–1.17)	<0.001
	W/O arthritis (*n* = 17,361)		1.06 (1.05–1.06)	<0.001
Spent ≥7 h per night sleeping in individuals who report passive smoke exposure	Arthritis (*n* = 5532)		1.02 (1.01–1.04)	0.003
	W/O arthritis (*n* = 17,346)		0.91 (0.91–0.92)	<0.001
Perceived general health in individuals who report passive smoke exposure	Arthritis (*n* = 6534)		0.69 (0.68–0.70)	<0.001
	W/O arthritis (*n* = 19,543)		0.69 (0.68–0.69)	<0.001
Perceived mental health in individuals who report passive smoke exposure	Arthritis (*n* = 6533)		0.64 (0.62–0.65)	<0.001
	W/O arthritis (*n* = 19,537)		0.76 (0.75–0.77)	<0.001
Mood disorder in individuals who report passive smoke exposure	Arthritis (*n* = 6535)		1.18 (1.16–1.20)	<0.001
	W/O arthritis (*n* = 19,545)		1.54 (1.53–1.56)	<0.001
Anxiety disorder in individuals who report passive smoke exposure	Arthritis (*n* = 6528)		1.61 (1.58–1.64)	<0.001
	W/O arthritis (*n* = 19,546)		1.36 (1.35–1.38)	<0.001
Perceived oral health in individuals who report passive smoke exposure	Arthritis (*n* = 5098)		0.73 (0.71–0.74)	<0.001
	W/O arthritis (*n* = 16,205)		0.75 (0.74–0.76)	<0.001
Mouth pain in individuals who report passive smoke exposure	Arthritis (*n* = 5093)		1.84 (1.80–1.87)	<0.001
	W/O arthritis (*n* = 16,202)		1.22 (1.21–1.23)	<0.001
Bleeding gums in individuals who report passive smoke exposure	Arthritis (*n* = 3785)		1.26 (1.24–1.28)	<0.001
	W/O arthritis (*n* = 14,692)		1.03 (1.02–1.04)	<0.001
Mouth dryness in individuals who report passive smoke exposure	Arthritis (*n* = 5093)		1.46 (1.44–1.48)	<0.001
	W/O arthritis (*n* = 16,188)		1.36 (1.35–1.37)	<0.001
Uncomfortable eating in individuals who report passive smoke exposure	Arthritis (*n* = 5091)		1.27 (1.25–1.29)	<0.001
	W/O arthritis (*n* = 16,198)		1.33 (1.32–1.34)	<0.001

Association of sleep quality, general health, mental health, and oral health with smoking and passive smoking in participants with and without arthritis.. Binary logistic regression adjusted for age, sex, BMI, smoking status, and drinking status. Data presented as odds ratios (ORs) and 95% confidence intervals; all analyses were weighted. Data considered statistically significant when *p*-value ≤ 0.05. W/O = without; OR = odds ratio; CI = confidence interval; *n* = number of respondents; ref = reference.

**Table 6:** Association of sleep quality with general, mental, and oral health in participants with and without arthritis.

Variables	Population	OR (95% CI)	*p*-Value
Refreshing sleep (ref: Sleep unrefreshing)			
Perceived General Health	Arthritis (*n* = 11,800)	2.96 (2.94–2.98)	<0.001
	W/O arthritis (*n* = 37,039)	3.57 (3.55–3.58)	<0.001
Perceived Mental Health	Arthritis (*n* = 11,791)	4.32 (4.28–4.36)	<0.001
	W/O arthritis (*n* = 37,080)	3.76 (3.74 –3.78)	<0.001
Perceived Oral Health	Arthritis (*n* = 5147)	2.19 (2.16–2.22)	<0.001
	W/O arthritis (*n* = 16,650)	2.48 (2.46–2.50)	<0.001
Difficulty staying awake (ref: No difficulty)			
Perceived General Health	Arthritis (*n* = 11,778)	0.53 (0.53–0.54)	<0.001
	W/O arthritis (*n* = 37,066)	0.55 (0.55–0.56)	<0.001
Perceived Mental Health	Arthritis (*n* = 11,772)	0.41 (0.40–0.41)	<0.001
	W/O arthritis (*n* = 37,054)	0.47 (0.46–0.47)	<0.001
Perceived Oral Health	Arthritis (*n* = 5139)	0.61 (0.60–0.62)	<0.001
	W/O arthritis (*n* = 16,657)	0.64 (0.64–0.65)	<0.001
Trouble going to sleep (ref: No trouble)			
Perceived General Health	Arthritis (*n* = 11,830)	0.58 (0.58–0.59)	<0.001
	W/O arthritis (*n* = 37,151)	0.39 (0.38–0.39)	<0.001
Perceived Mental Health	Arthritis (*n* = 11,825)	0.41 (0.41–0.42)	<0.001
	W/O arthritis (*n* = 37,138)	0.33 (0.33–0.33)	<0.001
Perceived Oral Health	Arthritis (*n* = 5163)	0.72 (0.72–0.73)	<0.001
	W/O arthritis (*n* = 16,687)	0.54 (0.53–0.54)	<0.001
Spent ≥7 h per night sleeping (ref: <7 h)			
Perceived General Health	Arthritis (*n* = 11,770)	1.59 (1.58–1.60)	<0.001
	W/O arthritis (*n* = 37,069)	1.37 (1.36–1.38)	<0.001
Perceived Mental Health	Arthritis (*n* = 11,765)	1.60 (1.59 –1.62)	<0.001
	W/O arthritis (*n* = 37,059)	1.54 (1.54–1.55)	<0.001
Perceived Oral Health	Arthritis (*n* = 5147)	1.69 (1.67–1.71)	<0.001
	W/O arthritis (*n* = 16,676)	1.66 (1.65–1.67)	<0.001

Association of sleep quality with general, mental, and oral health in participants with and without arthritis. Binary logistic regression adjusted for age, sex, BMI, smoking status, and drinking status. Data presented as odds ratios and 95% confidence intervals; all analyses were weighted. Data considered statistically significant when *p*-value ≤ 0.05. W/O = without; OR = odds ratio; CI = confidence interval; *n* = number of respondents; ref = reference.

## Data Availability

Canadian Community Health Survey microdata file is publicly available on the Statistics Canada website.

## Supplementary Materials

The following supporting information can be downloaded at: https://www.mdpi.com/article/10.3390/nu16132091/s1, Supplemental Table S1: Association between sleep quality, general and mental health with dietary intake in arthritis/non-arthritis participants, Model 1, 2, and 3; Supplemental Table S2: Association between sleep quality, general, mental, and oral health with smoking/passive smoking in arthritis/non-arthritis participants, Model 1, 2, and 3; Supplemental Table S3: Association between Perceived General and Mental and Oral Health with Sleep Quality in Participants with Arthritis and without Arthritis, Model 1, 2, and 3.

## Abbreviations

ACREU	Arthritis Community Research and Evaluation Unit
BMI	Body Mass Index
CCHS	Canadian Community Health Survey
CDC	Center for Disease Control and Prevention
CI	Confidence Interval
HFD	High-fat Diet
OA	Osteoarthritis
OR	Odd Ratio
RA	Rheumatoid Arthritis
