# Supplementary material for: Association of Sleep Quality and General, Mental, and Oral Health with Lifestyle Traits (Dietary Intake, Smoking Status) in Arthritis: A Cross-Sectional Study from the Canadian Community Health Survey (CCHS)
Source: Nutrients. 2024 Jun 29;16(13):2091. doi: 10.3390/nu16132091 (PMC11243648; doi:10.3390/nu16132091)
Supplement: Supplementary file 1 [file nutrients-16-02091-s001.zip › nutrients-3052978-supplementary.pdf]

Table S1: Association between sleep quality, general and mental health with dietary intake in arthritis/non-arthritis participants

Variables	Population	Models	OR (95% CI)	P-value
Self-reported food choice				
Choose for lower fat content (ref: not choosing)				
Refreshing sleep in individuals who choose lower fat content	Arthritis n= 3374	Model 1	1.51 (1.49-1.53)	<0.001
		Model 2	1.49 (1.47-1.51)	<0.001
		Model 3	1.51 (1.49-1.52)	<0.001
	W/O arthritis n= 10,319	Model 1	1.00 (0.99-1.01)	0.670
		Model 2	1.02 (1.01-1.03)	0.001
		Model 3	1.00 (0.99-1.01)	0.708
Difficulty to stay awake in individuals who choose lower fat content	Arthritis n= 3363	Model 1	1.15 (1.14-1.16)	<0.001
		Model 2	1.18 (1.17-1.20)	<0.001
		Model 3	1.21 (1.19-1.22)	<0.001
	W/O arthritis n= 10,285	Model 1	1.19 (1.18-1.19)	<0.001
		Model 2	1.23 (1.23-1.24)	<0.001
		Model 3	1.25 (1.24-1.25)	<0.001
Trouble to go to sleep in individuals who choose lower fat content	Arthritis n= 3379	Model 1	1.00 (0.99-1.01)	0.556
		Model 2	0.96 (0,95-0.97)	<0.001
		Model 3	0.95 (0.94-0.96)	<0.001
	W/O arthritis n= 10,330	Model 1	1.12 (1.11-1.12)	<0.001
		Model 2	1.08 (1.07-1.08)	<0.001
		Model 3	1.09 (1.08-1.09)	<0.001
Spent sleeping ≥ 7 hours per night in individuals who choose lower fat content	Arthritis n= 3348	Model 1	0.98 (0.98-1.00)	0.002
		Model 2	0.99 (0.99-1.00)	0.005
		Model 3	1.00 (0.99-1.01)	0.785
	W/O arthritis n= 10,285	Model 1	1.00 (0.99-1.00)	0.045
		Model 2	0.99 (0.98-0.99)	<0.001
		Model 3	0.96 (0.95-0.96)	<0.001
Perceived general health in individuals who choose lower fat content	Arthritis n= 3388	Model 1	1.13 (1.11-1.14)	<0.001
		Model 2	1.18 (1.17-1.20)	<0.001
		Model 3	1.12 (1.10-1.13)	<0.001
	W/O arthritis n= 10,329	Model 1	1.10 (1.09-1.11)	<0.001
		Model 2	1.20 (1.19-1.21)	<0.001
		Model 3	1.13 (1.12-1.14)	<0.001
Perceived mental health in individuals who choose lower fat content	Arthritis n= 3380	Model 1	1.32 (1.30-1.34)	<0.001
		Model 2	1.28 (1.26-1.30)	<0.001
		Model 3	1.18 (1.16-1.20)	<0.001
	W/O arthritis n= 10,323	Model 1	1.41 (1.40-1.42)	<0.001
		Model 2	1.39 (1.38-1.40)	<0.001
		Model 3	1.30 (1.29-1.31)	<0.001
Mood disorder in individuals who choose lower fat content	Arthritis n= 3384	Model 1	0.90 (0.89-0.91)	<0.001
		Model 2	0.95 (0.94-0.96)	<0.001
		Model 3	1.04 (1.02-1.05)	<0.001
		Model 1	0.86 (0.86-0.87)	<0.001

	W/O arthritis n= 10,325	Model 2	0.84 (0.83-0.85)	<0.001
		Model 3	0.88 (0.87-0.88)	<0.001
Anxiety disorder in individuals who choose lower fat content (ref: no anxiety disorder)	Arthritis n= 3382	Model 1	0.81 (0.80-0.82)	<0.001
		Model 2	0.89 (0.87-0.90)	<0.001
		Model 3	0.94 (0.93-0.96)	<0.001
	W/O arthritis n= 10,326	Model 1	0.86 (0.85-0.87)	<0.001
		Model 2	0.87 (0.86-0.87)	<0.001
		Model 3	0.92 (0.91-0.93)	<0.001
Choose for fiber content (ref: not choosing)				
Refreshing sleep in individual who choose fiber content (ref: not choosing)	Arthritis n= 3379	Model 1	1.17 (1.16-1.18)	<0.001
		Model 2	1.16 (1.14-1.17)	<0.001
		Model 3	1.15 (1.14-1.17)	<0.001
	W/O arthritis n= 10,312	Model 1	1.17 (1.16-1.17)	<0.001
		Model 2	1.30 (1.17-1.45)	<0.001
		Model 3	1.18 (1.18-1.19)	<0.001
Difficulty to stay awake in individual who choose fiber content (ref: not choosing)	Arthritis n= 3366	Model 1	1.27 (1.25-1.28)	<0.001
		Model 2	1.28 (1.26-1.29)	<0.001
		Model 3	1.30 (1.28-1.31)	<0.001
	W/O arthritis n= 10,278	Model 1	1.16 (1.16-1.17)	<0.001
		Model 2	1.24 (1.24-1.25)	<0.001
		Model 3	1.25 (1.25-1.26)	<0.001
Trouble to go to sleep in individual who choose fiber content (ref: not choosing)	Arthritis n= 3383	Model 1	1.24 (1.23-1.25)	<0.001
		Model 2	1.13 (1.11-1.14)	<0.001
		Model 3	1.12 (1.11-1.14)	<0.001
	W/O arthritis n= 10,323	Model 1	1.11 (1.10-1.11)	<0.001
		Model 2	1.04 (1.03-1.04)	<0.001
		Model 3	1.04 (1.04-1.05)	<0.001
Spent sleeping ≥ 7 hours per night in individual who choose fiber content (ref: not choosing)	Arthritis n= 3351	Model 1	1.00 (0.99-1.02)	0.429
		Model 2	0.98 (0.97-0.99)	<0.001
		Model 3	1.01 (1.00-1.02)	0.064
	W/O arthritis n= 10,278	Model 1	1.10 (1.10-1.11)	<0.001
		Model 2	1.09 (1.09-1.10)	<0.001
		Model 3	1.06 (1.05-1.06)	<0.001
Perceived general health in individual who choose fiber content (ref: not choosing)	Arthritis n= 3393	Model 1	1.06 (1.05-1.07)	<0.001
		Model 2	1.11 (1.10-1.13)	<0.001
		Model 3	1.06 (1.04-1.07)	<0.001
	W/O arthritis n= 10,323	Model 1	1.07 (1.06-1.08)	<0.001
		Model 2	1.23 (1.22-1.24)	<0.001
		Model 3	1.11 (1.10-1.12)	<0.001
Perceived mental health in individual who choose fiber content (ref: not choosing)	Arthritis n= 3384	Model 1	1.12 (1.10-1.13)	<0.001
		Model 2	1.04 (1.02-1.05)	<0.001
		Model 3	0.96 (0.95-0.98)	<0.001
	W/O arthritis n= 10,315	Model 1	1.67 (1.66-1.69)	<0.001
		Model 2	1.66 (1.64-1.67)	<0.001
		Model 3	1.54 (1.52-1.55)	<0.001

Mood disorder in individual who choose fiber content (ref: not choosing)	Arthritis n= 3388	Model 1	1.09 (1.07-1.11)	<0.001
		Model 2	1.13 (1.11-1.15)	<0.001
		Model 3	1.22 (1.20-1.24)	<0.001
	W/O arthritis n= 10,318	Model 1	0.85 (0.84-0.86)	<0.001
		Model 2	0.81 (0.80-0.813)	<0.001
		Model 3	0.88 (0.88-0.89)	<0.001
Anxiety disorder in individual who choose fiber content (ref: not choosing)	Arthritis n= 3386	Model 1	0.89 (0.87-0.90)	<0.001
		Model 2	0.98 (0.96-0.99)	0.007
		Model 3	1.03 (1.01-1.05)	0.001
	W/O arthritis n= 10,319	Model 1	0.85 (0.84-0.85)	<0.001
		Model 2	0.87 (0.86-0.87)	<0.001
		Model 3	0.94 (0.93-0.95)	<0.001
Avoid for cholesterol content (ref: not avoiding)				
Refreshing sleep in individual who avoid cholesterol content (ref: not avoiding)	Arthritis n= 3367	Model 1	1.34 (1.33-1.36)	<0.001
		Model 2	1.31 (1.30-1.33)	<0.001
		Model 3	1.24 (1.23-1.26)	<0.001
	W/O arthritis n= 10,299	Model 1	1.23 (1.22-1.23)	<0.001
		Model 2	1.22 (1.21-1.23)	<0.001
		Model 3	1.19 (1.18-1.20)	<0.001
Difficulty to stay awake in individual who avoid cholesterol content (ref: not avoiding)	Arthritis n= 3356	Model 1	1.32 (1.30-1.33)	<0.001
		Model 2	1.34 (1.33-1.36)	<0.001
		Model 3	1.26 (1.24 -1.27)	<0.001
	W/O arthritis n= 10,267	Model 1	1.18 (1.17-1.19)	<0.001
		Model 2	1.25 (1.25-1.26)	<0.001
		Model 3	1.25 (1.24-1.16)	<0.001
Trouble to go to sleep in individual who avoid cholesterol content (ref: not avoiding)	Arthritis n= 3373	Model 1	1.11 (1.10-1.12)	<0.001
		Model 2	1.09 (1.08-1.10)	<0.001
		Model 3	1.14 (1.13-1.16)	<0.001
	W/O arthritis n= 10,310	Model 1	1.09 (1.18-1.09)	<0.001
		Model 2	1.08 (1.08-1.09)	<0.001
		Model 3	1.13 (1.12-1.13)	<0.001
Spent sleeping ≥ 7 hours per night in individual who avoid cholesterol content (ref: not avoiding)	Arthritis n= 3341	Model 1	0.84 (0.83-0.85)	<0.001
		Model 2	0.83 (0.82-0.84)	<0.001
		Model 3	0.88 (0.87-0.89)	<0.001
	W/O arthritis n= 10,265	Model 1	0.85 (0.85-0.85)	<0.001
		Model 2	0.84 (0.83-0.84)	<0.001
		Model 3	0.83 (0.83-0.84)	<0.001
Perceived general health in individual who avoid cholesterol content (ref: not avoiding)	Arthritis n= 3382	Model 1	0.97 (0.96-0.98)	<0.001
		Model 2	0.99 (0.98-1.00)	<0.001
		Model 3	1.00 (0.99-1.02)	0.519
	W/O arthritis n= 10,309	Model 1	0.87 (0.87-0.88)	<0.001
		Model 2	0.96 (0.96-0.97)	<0.001
		Model 3	0.91 (0.91-0.92)	<0.001
Perceived mental health in individual who avoid cholesterol content	Arthritis n= 3374	Model 1	1.07 (1.05-1.08)	<0.001
		Model 2	1.03 (1.02-1.05)	<0.001
		Model 3	0.99 (0.97-1.00)	0.063

(ref: not avoiding)	W/O arthritis n= 10,302	Model 1	1.38 (1.37-1.39)	<0.001
		Model 2	1.31 (1.30-1.32)	<0.001
		Model 3	1.25 (1.24-1.27)	<0.001
Mood disorder in individual who avoid cholesterol content (ref: not avoiding)	Arthritis n= 3378	Model 1	0.91 (0.89-0.92)	<0.001
		Model 2	0.95 (0.94-0.97)	<0.001
		Model 3	0.97 (0.95-0.98)	<0.001
	W/O arthritis n= 10,306	Model 1	0.77 (0.77-0.78)	<0.001
		Model 2	0.80 (0.79-0.80)	<0.001
		Model 3	0.85 (0.84-0.86)	<0.001
Anxiety disorder in individual who avoid cholesterol content (ref: not avoiding)	Arthritis n= 3376	Model 1	1.16 (1.15-1.18)	<0.001
		Model 2	1.30 (1.28-1.32)	<0.001
		Model 3	1.32 (1.30-1.34)	<0.001
	W/O arthritis n= 10,307	Model 1	0.77 (0.76-0.77)	<0.001
		Model 2	0.83 (0.83-0.84)	<0.001
		Model 3	0.92 (0.910-0.93)	<0.001
**Avoid for calorie content (ref: not avoiding)**				
**Refreshing sleep** in individual who avoid calorie content (ref: not avoiding)	Arthritis n= 3376	Model 1	1.20 (1.18-1.21)	<0.001
		Model 2	1.27 (1.25-1.28)	<0.001
		Model 3	1.23 (1.22-1.25)	<0.001
	W/O arthritis n= 10,315	Model 1	0.95 (0.94-0.95)	<0.001
		Model 2	1.01 (1.01-1.02)	<0.001
		Model 3	1.03 (1.03-1.04)	<0.001
**Difficulty to stay awake** in individual who avoid calorie content (ref: not avoiding)	Arthritis n= 3363	Model 1	1.22 (1.21-1.23)	<0.001
		Model 2	1.19(1.17-1.20)	<0.001
		Model 3	1.14 (1.13-1.16)	<0.001
	W/O arthritis n= 10,282	Model 1	1.13 (1.12-1.13)	<0.001
		Model 2	1.12 (1.11-1.13)	<0.001
		Model 3	1.17 (1.16-1.17)	<0.001
**Trouble to go to sleep** in individual who avoid calorie content (ref: not avoiding)	Arthritis n= 3379	Model 1	1.17 (1.16-1.19)	<0.001
		Model 2	1.06 (1.05-1.07)	<0.001
		Model 3	1.07 (1.06-1.08)	<0.001
	W/O arthritis n= 10,326	Model 1	1.40 (1.39-1.40)	<0.001
		Model 2	1.31 (1.31-1.32)	<0.001
		Model 3	1.33 (1.32-1.33)	<0.001
**Spent sleeping ≥ 7 hours per night** in individual who avoid calorie content (ref: not avoiding)	Arthritis n= 3348	Model 1	0.96 (0.95-0.97)	<0.001
		Model 2	0.96 (0.95-0.97)	<0.001
		Model 3	1.00 (0.98-1.01)	0.301
	W/O arthritis n= 10,281	Model 1	0.94 (0.93-0.94)	<0.001
		Model 2	0.94 (0.94-0.95)	<0.001
		Model 3	0.94 (0.93-0.94)	<0.001
**Perceived general health** in individual who avoid calorie content (ref: not avoiding)	Arthritis n= 3387	Model 1	1.20 (1.19-1.21)	<0.001
		Model 2	1.24 (1.23-1.26)	<0.001
		Model 3	1.20 (1.18-1.21)	<0.001
	W/O arthritis n= 10,324	Model 1	1.41 (1.40-1.42)	<0.001
		Model 2	1.44 (1.43-1.46)	<0.001
		Model 3	1.38 (1.37-1.39)	<0.001

**Perceived mental health** in individual who avoid calorie content (ref: not avoiding)	Arthritis n= 3379	Model 1	1.39 (1.37-1.41)	<0.001
		Model 2	1.39 (1.37-1.41)	<0.001
		Model 3	1.28 (1.26-1.30)	<0.001
	W/O arthritis n= 10,318	Model 1	1.41 (1.40-1.42)	<0.001
		Model 2	1.48 (1.47-1.49)	<0.001
		Model 3	1.38 (1.37-1.40)	<0.001
**Mood disorder** in individual who avoid calorie content (ref: not avoiding).	Arthritis n= 3383	Model 1	0.90 (0.88-0.91)	<0.001
		Model 2	0.85 (0.84-0.86)	<0.001
		Model 3	0.91 (0.90-0.93)	<0.001
	W/O arthritis n= 10,320	Model 1	0.93 (0.93-0.94)	<0.001
		Model 2	0.84 (0.83-0.85)	<0.001
		Model 3	0.83 (0.83-0.84)	<0.001
**Anxiety disorder** in individual who avoid calorie content (ref: not avoiding)	Arthritis n= 3381	Model 1	1.10 (1.08-1.11)	<0.001
		Model 2	1.07 (1.05-1.09)	<0.001
		Model 3	1.17 (1.15-1.19)	<0.001
	W/O arthritis n= 10,321	Model 1	0.97 (0.96-0.98)	<0.001
		Model 2	0.88 (0.87-0.89)	<0.001
		Model 3	0.87 (0.86-0.88)	<0.001

Table S1. Association between sleep quality, general health and mental health with dietary intake in arthritis and non-arthritis participants. Model 1: non-adjusted binary logistic regression, Model 2: binary logistic regression adjusted for age and sex, Model 3: binary logistic regression adjusted for age, sex, BMI, smoking status and drinking status. Data presented as odds ratio (OR) and 95% confidence intervals; all analysis were weighted. Data considered as statistically significant when p-value ≤ 0.05. W/O= without, OR= Odds Ratio, CI= Confidence Interval, n= number of respondents, ref= reference.

Table S2: Association between sleep quality, general, mental, and oral health with smoking/passive smoking in arthritis/non-arthritis participants

Variables	Population		Models	OR (95% CI)	P-value
Smokers (ref: never smoker)					
Refreshing sleep in individuals who are a current or former smoker	Arthritis n= 12,231	Current	Model 1	0.50 (0.49-0.50)	<0.001
			Model 2	0.54 (0.54-0.55)	<0.001
			Model 3	0.54 (0.53-0.54)	<0.001
		Former	Model 1	0.92 (0.91-0.92)	<0.001
			Model 2	0.81 (0.80-0.82)	<0.001
			Model 3	0.79 (0.78-0.80)	<0.001
	W/O arthritis n= 38,558	Current	Model 1	0.66 (0.66-0.66)	<0.001
			Model 2	0.63 (0.63-0.64)	<0.001
			Model 3	0.63 (0.63-0.63)	<0.001
		Former	Model 1	0.96 (0.95-0.96)	<0.001
			Model 2	0.85 (0.85-0.85)	<0.001
			Model 3	0.85 (0.84-0.85)	<0.001
Difficulty to stay awake in individuals who are a current or former smoker	Arthritis n= 12,207	Current	Model 1	1.15 (1.14-1.16)	<0.001
			Model 2	1.11 (1.10-1.12)	<0.001
			Model 3	1.14 (1.13-1.15)	<0.001
		Former	Model 1	0.90 (0.89-0.90)	<0.001
			Model 2	0.93 (0.93-0.94)	<0.001
			Model 3	0.94 (0.93-0.94)	<0.001
	W/O arthritis n= 38,510	Current	Model 1	1.050 (1.05-1.05)	<0.001
			Model 2	1.06 (1.06-1.07)	<0.001
			Model 3	1.08 (1.08-1.08)	<0.001
		Former	Model 1	0.84 (0.84-0.85)	<0.001
			Model 2	0.92 (0.91-0.92)	<0.001
			Model 3	0.92 (0.92-0.92)	<0.001
Trouble to go to sleep in individuals who are a current or former smoker	Arthritis n= 12,266	Current	Model 1	0.98 (0.97-0.98)	<0.001
			Model 2	0.99 (0.98-0.99)	<0.001
			Model 3	0.99 (0.98-0.99)	<0.001
		Former	Model 1	0.99 (0.98-01.00)	<0.001
			Model 2	1.13 (1.13-1.14)	<0.001
			Model 3	1.13 (1.12-1.14)	<0.001
	W/O arthritis n= 38,622	Current	Model 1	1.25 (1.25-1.26)	<0.001
			Model 2	1.36 (1.35-1.36)	<0.001
			Model 3	1.33 (1.32-1.33)	<0.001
		Former	Model 1	1.12 (1.12-1.12)	<0.001
			Model 2	1.21 (1.21-1.21)	<0.001
			Model 3	1.18 (1.17-1.18)	<0.001
Spent sleeping ≥ 7 hours per night in individuals who are a current or former smoker	Arthritis n= 12,194	Current	Model 1	0.84 (0.83-0.84)	<0.001
			Model 2	0.89 (0.89-0.90)	<0.001
			Model 3	0.91 (0.90-0.92)	<0.001
		Former	Model 1	1.08 (1.07-1.08)	<0.001
			Model 2	1.05 (1.05-1.06)	<0.001
			Model 3	1.05 (1.04-1.05)	<0.001
			Model 1	0.78 (0.78-0.79)	<0.001

	W/O arthritis n= 38,510	Current	Model 2	0.81 (0.80-0.81)	<0.001
			Model 3	0.80 (0.79-0.80)	<0.001
		Former	Model 1	0.99 (0.98-0.99)	<0.001
			Model 2	0.97 (0.97-0.97)	<0.001
			Model 3	0.97 (0.97-0.98)	<0.001
Perceived General health in individuals who are a current or former smoker	Arthritis n= 27,342	Current	Model 1	0.47 (0.47-0.47)	<0.001
			Model 2	0.45 (0.45-0.45)	<0.001
			Model 3	0.41 (0.40-0.41)	<0.001
		Former	Model 1	0.84 (0.84-0.85)	<0.001
			Model 2	0.85 (0.85-0.85)	<0.001
			Model 3	0.76 (0.76-0.77)	<0.001
	W/O arthritis n= 76,433	Current	Model 1	0.40 (0.40-0.40)	<0.001
			Model 2	0.39 (0.39-0.39)	<0.001
			Model 3	0.35 (0.34-0.35)	<0.001
		Former	Model 1	0.61 (0.61-0.62)	0.04
			Model 2	0.73 (0.73-0.74)	<0.001
			Model 3	0.68 (0.68-0.68)	<0.001
Perceived mental health in individuals who are a current or former smoker	Arthritis n= 26,355	Current	Model 1	0.37 (0.37-0.37)	<0.001
			Model 2	0.42 (0.42-0.42)	<0.001
			Model 3	0.41 (0.40-0.41)	<0.001
		Former	Model 1	0.97 (0.96-0.97)	<0.001
			Model 2	0.89 (0.89-0.90)	<0.001
			Model 3	0.84 (0.83-0.84)	<0.001
	W/O arthritis n= 74,882	Current	Model 1	0.44 (0.44-0.44)	<0.001
			Model 2	0.41 (0.41-0.41)	<0.001
			Model 3	0.40 (0.40-0.41)	<0.001
		Former	Model 1	1.04 (1.03-1.04)	0.04
			Model 2	0.83 (0.83-0.84)	<0.001
			Model 3	0.84 (0.84-0.84)	<0.001
Mood disorder in individuals who are a current or former smoker	Arthritis n= 27,351	Current	Model 1	2.95 (2.93-2.96)	<0.001
			Model 2	2.71 (2.69-2.72)	<0.001
			Model 3	2.88 (2.86-2.89)	<0.001
		Former	Model 1	1.18 (1.17-1.19)	<0.001
			Model 2	1.35 (1.34-1.36)	<0.001
			Model 3	1.43 (1.42-1.44)	<0.001
	W/O arthritis n= 76,401	Current	Model 1	2.44 (2.44-2.45)	<0.001
			Model 2	2.75 (2.74-2.76)	<0.001
			Model 3	2.73 (2.72-2.74)	<0.001
		Former	Model 1	1.35 (1.35-1.36)	<0.001
			Model 2	1.65 (1.65-1.66)	<0.001
			Model 3	1.58 (1.57-1.59)	<0.001
Anxiety disorder in individuals who are a current or former smoker	Arthritis n= 27,335	Current	Model 1	3.00 (2.98-3.02)	<0.001
			Model 2	2.74 (2.72-2.76)	<0.001
			Model 3	2.87 (2.85-2.89)	<0.001
(ref: never smoker)		Former	Model 1	1.04 (1.04-1.05)	<0.001
			Model 2	1.22 (1.21-1.23)	<0.001
			Model 3	1.29 (1.72-1.74)	<0.001
			Model 1	2.22 (2.21-2.23)	<0.001

	W/O arthritis n= 76,396	Current	Model 2	2.59 (2.58-2.60)	<0.001
			Model 3	2.62 (2.61-2.63)	<0.001
		Former	Model 1	1.15 (1.15-1.16)	<0.001
			Model 2	1.63 (1.62-1.64)	<0.001
			Model 3	1.63 (1.63-1.64)	<0.001
Perceived oral health in individuals who are a current or former smoker	Arthritis n= 13,482	Current	Model 1	0.34 (0.34-0.34)	<0.001
			Model 2	0.37 (0.37-0.37)	<0.001
			Model 3	0.36 (0.36-0.36)	<0.001
		Former	Model 1	1.00 (0.99-1.00)	0.325
			Model 2	1.02 (1.01-1.03)	<0.001
			Model 3	0.93 (0.92-0.94)	<0.001
	W/O arthritis n= 38,865	Current	Model 1	0.28 (0.28-0.28)	<0.001
			Model 2	0.29 (0.29-0.29)	<0.001
			Model 3	0.27 (0.27-0.27)	<0.001
		Former	Model 1	0.70 (0.69-0.70)	<0.001
			Model 2	0.74 (0.73-0.74)	<0.001
			Model 3	0.68 (0.68-0.68)	<0.001
Mouth pain in individuals who are a current or former smoker	Arthritis n= 13,489	Current	Model 1	1.60 (1.59-1.62)	<0.001
			Model 2	1.48 (1.46-1.49)	<0.001
			Model 3	1.47 (1.46-1.48)	<0.001
		Former	Model 1	0.93 (0.93-0.94)	<0.001
			Model 2	1.04 (1.03-1.05)	<0.001
			Model 3	1.11 (1.10-1.11)	<0.001
	W/O arthritis n= 38,871	Current	Model 1	1.71 (1.71-1.72)	<0.001
			Model 2	1.79 (1.79-1.80)	<0.001
			Model 3	1.81 (1.80-1.82)	<0.001
		Former	Model 1	1.05 (1.05-1.06)	<0.001
			Model 2	1.18 (1.18-1.19)	<0.001
			Model 3	1.22 (1.22-1.23)	<0.001
Bleeding gums in individuals who are a current or former smoker	Arthritis n= 11,074	Current	Model 1	0.97 (0.96-0.98)	<0.001
			Model 2	0.74 (0.74-0.75)	<0.001
			Model 3	0.71 (0.71-0.72)	<0.001
		Former	Model 1	0.87 (0.87-0.88)	<0.001
			Model 2	0.96 (0.95-0.97)	<0.001
			Model 3	1.05 (1.04-1.06)	<0.001
	W/O arthritis n= 36,254	Current	Model 1	1.01 (1.01-1.02)	<0.001
			Model 2	1.01 (1.00-1.01)	<0.001
			Model 3	1.01 (1.00-1.01)	0.009
		Former	Model 1	0.83 (0.83-0.83)	<0.001
			Model 2	1.060 (1.06-1.06)	<0.001
			Model 3	1.03 (1.02-1.03)	<0.001
Mouth dryness in individuals who are a current or former smoker	Arthritis n= 13,478	Current	Model 1	1.76 (1.75-1.77)	<0.001
			Model 2	1.97 (1.96-1.98)	<0.001
			Model 3	2.03 (2.02-2.04)	<0.001
		Former	Model 1	1.11 (1.11-1.12)	<0.001
			Model 2	1.16 (1.15-1.16)	<0.001
			Model 3	1.24 (1.23-1.25)	<0.001
			Model 1	1.95 (1.94-1.96)	<0.001

	W/O arthritis n= 38,826	Current	Model 2	2.00 (1.99-2.00)	<0.001
			Model 3	2.08 (2.07-2.08)	<0.001
		Former	Model 1	1.52 (1.52-1.53)	<0.001
			Model 2	1.37 (1.36-1.37)	<0.001
			Model 3	1.09 (1.09-1.10)	<0.001
Uncomfortable to eat in individuals who are a current or former smoker	Arthritis n= 13,483	Current	Model 1	1.77 (1.76-1.79)	<0.001
			Model 2	1.71 (1.70-1.73)	<0.001
			Model 3	1.65 (1.63-1.66)	<0.001
		Former	Model 1	1.00 (0.99-1.00)	0.113
			Model 2	1.09 (1.08-1.09)	<0.001
			Model 3	1.15 (1.14-1.15)	<0.001
	W/O arthritis n= 38,870	Current	Model 1	1.83 (1.82-1.83)	<0.001
			Model 2	1.92 (1.91-1.92)	<0.001
			Model 3	1.94 (1.93-1.95)	<0.001
		Former	Model 1	1.25 (1.24-1.25)	<0.001
			Model 2	1.34 (1.33-1.34)	<0.001
			Model 3	1.39 (1.39-1.40)	<0.001
Passive smokers (ref: not passive smoker)					
Refreshing sleep in individuals who are being passive smoker		Arthritis n= 5689	Model 1	0.61 (0.60-0.62)	<0.001
			Model 2	0.65 (0.65-0.66)	<0.001
			Model 3	0.85 (0.83-0.86)	<0.001
		W/O arthritis n= 17,899	Model 1	0.73 (0.72-0.73)	<0.001
			Model 2	0.72 (0.72-0.73)	<0.001
			Model 3	0.94 (0.93-0.95)	<0.001
Difficulty to stay awake in individuals who are being passive smoker		Arthritis n= 5678	Model 1	1.16 (1.14-1.17)	<0.001
			Model 2	1.12 (1.11-1.14)	<0.001
			Model 3	1.12 (1.10-1.13)	<0.001
		W/O arthritis n= 17,896	Model 1	1.11 (1.11-1.12)	0.001
			Model 2	1.12 (1.11-1.13)	<0.001
			Model 3	1.07 (1.06-1.08)	<0.001
Trouble to go to sleep in individuals who are being passive smoker		Arthritis n= 5706	Model 1	1.02 (1.01-1.03)	0.004
			Model 2	0.96 (0.94-0.97)	<0.001
			Model 3	1.15 (1.13-1.17)	<0.001
		W/O arthritis n= 17,935	Model 1	1.15 (1.14-1.15)	<0.001
			Model 2	1.16 (1.15-1.17)	<0.001
			Model 3	1.06 (1.05-1.06)	<0.001
Spent sleeping ≥ 7 hours per in individuals who are being passive smoker		Arthritis n= 5687	Model 1	0.99 (0.97-1.00)	0.008
			Model 2	1.00 (0.99-1.01)	0.655
			Model 3	1.02 (1.01-1.04)	0.003
		W/O arthritis n= 17,911	Model 1	0.79 (0.78-0.79)	<0.001
			Model 2	0.79 (0.78-0.79)	<0.001
			Model 3	0.91 (0.91-0.92)	<0.001
Perceived General health in individuals who are being passive smoker		Arthritis n= 6942	Model 1	0.51 (0.50-0.51)	<0.001
			Model 2	0.49 (0.48-0.49)	<0.001
			Model 3	0.69 (0.68-0.70)	<0.001
		W/O arthritis	Model 1	0.42 (0.42-0.43)	<0.001
			Model 2	0.42 (0.42-0.43)	<0.001

	n= 20,559	Model 3	0.69 (0.68-0.69)	<0.001
Perceived mental health in individuals who are being passive smoker	Arthritis n= 6734	Model 1	0.38 (0.37-0.38)	<0.001
		Model 2	0.40 (0.40-0.41)	<0.001
		Model 3	0.64 (0.62-0.65)	<0.001
	W/O arthritis n= 20,200	Model 1	0.49 (0.48-0.49)	<0.001
		Model 2	0.48 (0.48-0.49)	<0.001
		Model 3	0.76 (0.75-0.77)	<0.001
Mood disorder in individuals who are being passive smoker	Arthritis n= 6942	Model 1	2.19 (2.15-2.22)	<0.001
		Model 2	2.00 (1.97-2.03)	<0.001
		Model 3	1.18 (1.16-1.20)	<0.001
	W/O arthritis n= 20,558	Model 1	2.31 (2.29-2.33)	<0.001
		Model 2	2.37 (2.35-2.40)	<0.001
		Model 3	1.54 (1.53-1.56)	<0.001
Anxiety disorder in individuals who are being passive smoker	Arthritis n= 6937	Model 1	2.48 (2.45-2.52)	<0.001
		Model 2	2.36 (2.33-2.39)	<0.001
		Model 3	1.61 (1.58-1.64)	<0.001
	W/O arthritis n= 20,556	Model 1	1.79 (1.77-1.80)	<0.001
		Model 2	1.85 (1.83-1.86)	<0.001
		Model 3	1.36 (1.35-1.38)	<0.001
Perceived oral health in individuals who are being passive smoker	Arthritis n= 5237	Model 1	0.44 (0.43-0.45)	<0.001
		Model 2	0.44 (0.43-0.45)	<0.001
		Model 3	0.73 (0.71-0.74)	0.001
	W/O arthritis n= 16,718	Model 1	0.41 (0.41-0.41)	<0.001
		Model 2	0.41 (0.41-0.42)	<0.001
		Model 3	0.75 (0.74-0.76)	<0.001
Mouth pain in individuals who are being passive smoker	Arthritis n= 5234	Model 1	1.86 (1.83-1.88)	<0.001
		Model 2	1.76 (1.74-1.79)	<0.001
		Model 3	1.84 (1.80-1.87)	<0.001
	W/O arthritis n= 16,714	Model 1	1.51 (1.50-1.52)	<0.001
		Model 2	1.54 (1.53-1.56)	<0.001
		Model 3	1.22 (1.21-1.23)	<0.001
Bleeding gums in individuals who are being passive smoker	Arthritis n= 3886	Model 1	1.10 (1.09-1.12)	<0.001
		Model 2	0.95 (0.94-0.97)	<0.001
		Model 3	1.26 (1.24-1.28)	<0.001
	W/O arthritis n= 15,167	Model 1	1.00 (0.99-1.01)	0.595
		Model 2	0.99 (0.99-1.00)	0.068
		Model 3	1.03 (1.02-1.04)	<0.001
Mouth dryness in individuals who are being passive smoker	Arthritis n= 5232	Model 1	1.80 (1.78-1.82)	<0.001
		Model 2	1.90 (1.88-1.92)	<0.001
		Model 3	1.46 (1.44-1.48)	<0.001
	W/O arthritis n= 16,699	Model 1	1.85 (1.84-1.86)	<0.001
		Model 2	1.84 (1.83-1.85)	<0.001
		Model 3	1.36 (1.35-1.37)	<0.001
Uncomfortable to eat in individuals who are being passive smoker	Arthritis n= 5232	Model 1	1.58 (1.56-1.60)	<0.001
		Model 2	1.51 (1.49-1.53)	<0.001
		Model 3	1.27 (1.25-1.29)	<0.001
		Model 1	1.60 (1.59-1.61)	<0.001

	W/O arthritis n= 16.710	Model 2	1.65 (1.64-1.66)	<0.001
		Model 3	1.33 (1.32-1.34)	<0.001

Table S2. Association between sleep quality, general health, mental health and oral health with smoking and passive smoking in arthritis and non-arthritis participants. Model 1: non-adjusted binary logistic regression, Model 2: binary logistic regression adjusted for age and sex, Model 3: binary logistic regression adjusted for age, sex, BMI, smoking status and drinking status. Data presented as odds ratio (OR) and 95% confidence intervals; all analysis were weighted. Data considered as statistically significant when p-value ≤ 0.05. W/O= without, OR= Odds Ratio, CI= Confidence Interval, n= number of respondents, ref= reference.

Supplemental Table S3: Association between Perceived General and Mental and Oral Health with Sleep Quality in Participants with Arthritis and Without Arthritis

Variables	Population	Models	OR (95% CI)	P-value
Refreshing sleep (ref: Sleep unrefreshing)				
Perceived General Health	Arthritis n= 12,261	Model 1	3.10 (3.08-3.12)	<0.001
		Model 2	3.24 (3.22-3.27)	<0.001
		Model 3	2.96 (2.94-2.98)	<0.001
	W/O arthritis n= 38,605	Model 1	3.65 (3.63-3.67)	0.670
		Model 2	4.00 (3.98-4.02)	0.001
		Model 3	3.57 (3.55-3.58)	0.708
Perceived Mental Health	Arthritis n= 12,251	Model 1	5.22 (5.17-5.26)	<0.001
		Model 2	4.69 (4.46-4.73)	<0.001
		Model 3	4.32 (4.28-4.36)	<0.001
	W/O arthritis n= 38,587	Model 1	4.22 (4.20-4.25)	<0.001
		Model 2	4.08 (4.06-4.10)	<0.001
		Model 3	3.76 (3.74 -3.78)	<0.001
Perceived Oral Health	Arthritis n= 5292	Model 1	2.58 (2.55-2.61)	0.556
		Model 2	2.57 (2.54-2.61)	<0.001
		Model 3	2.19 (2.16-2.22)	<0.001
	W/O arthritis n=17,223	Model 1	2.81 (2.79-2.83)	<0.001
		Model 2	2.86 (2.73-2.88)	<0.001
		Model 3	2.48 (2.46-2.50)	<0.001
Difficulty to stay awake (ref: No difficulty)				
Perceived General Health	Arthritis n= 12.236	Model 1	0.52 (0.52-0.523)	<0.001
		Model 2	0.52 (0.52-0.52)	<0.001
		Model 3	0.53 (0.53-0.54)	<0.001
	W/O arthritis n=38,557	Model 1	0.57 (0.56-0.57)	<0.001
		Model 2	0.54 (0.54-0.54)	<0.001
		Model 3	0.55 (0.55-0.56)	<0.001
Perceived Mental Health	Arthritis n= 12,230	Model 1	0.39 (0.39-0.39)	<0.001
		Model 2	0.40 (0.40-0.41)	<0.001
		Model 3	0.41 (0.40-0.41)	<0.001
	W/O arthritis n=38,542	Model 1	0.44 (0.44-0.44)	<0.001
		Model 2	0.46 (0.46-0.46)	<0.001
		Model 3	0.47 (0.46-0.47)	<0.001
Perceived Oral Health	Arthritis n= 5281	Model 1	0.63 (0.62-0.63)	<0.001
		Model 2	0.63 (0.62-0.64)	<0.001
		Model 3	0.61 (0.60-0.62)	<0.001
	W/O arthritis n=17,229	Model 1	0.59 (0.59-0.60)	<0.001
		Model 2	0.59 (0.59-0.60)	<0.001
		Model 3	0.64 (0.64-0.65)	<0.001
Trouble to go to sleep (ref: No trouble)				
Perceived General Health	Arthritis n= 12,293	Model 1	0.62 (0.61-0.62)	<0.001
		Model 2	0.61 (0.60-0.61)	<0.001
		Model 3	0.58 (0.58-0.59)	<0.001
	W/O arthritis	Model 1	0.40 (0.39-0.40)	<0.001
		Model 2	0.58 (0.58-0.58)	<0.001

	n=38,666	Model 3	0.39 (0.38-0.39)	<0.001
Perceived Mental Health	Arthritis	Model 1	0.41 (0.41-0.42)	<0.001
	n= 12,288	Model 2	0.42 (0.42-0.43)	<0.001
		Model 3	0.41 (0.41-0.42)	<0.001
	W/O arthritis	Model 1	0.32 (0.32-0.32)	<0.001
	n=38,649	Model 2	0.32 (0.32-0.33)	<0.001
		Model 3	0.33 (0.33-0.33)	<0.001
Perceived Oral Health	Arthritis	Model 1	0.84 (0.83-0.85)	<0.001
	n= 5308	Model 2	0.80 (0.79-0.81)	<0.001
		Model 3	0.72 (0.72-0.73)	<0.001
	W/O arthritis	Model 1	0.55 (0.55-0.56)	<0.001
	n=17.265	Model 2	0.52 (0.52-0.53)	<0.001
		Model 3	0.54 (0.53-0.54)	<0.001
Spent sleeping ≥ 7 hours per night (ref: < 7 hours)				
Perceived General Health	Arthritis	Model 1	1.71 (1.70-1.72)	<0.001
	n= 12,223	Model 2	1.72 (1.71-1.73)	<0.001
		Model 3	1.59 (1.58-1.60)	<0.001
	W/O arthritis	Model 1	1.48 (1.47-1.48)	<0.001
	n= 38,551	Model 2	1.53 (1.52-1.53)	<0.001
		Model 3	1.37 (1.36-1.38)	<0.001
Perceived Mental Health	Arthritis	Model 1	1.82 (1.80 -1.83)	<0.001
	n= 12,218	Model 2	1.69 (1.68 -1.71)	<0.001
		Model 3	1.60 (1.59 -1.62)	<0.001
	W/O arthritis	Model 1	1.66 (1.65-1.67)	<0.001
	n=38,539	Model 2	1.66 (1.65-1.66)	<0.001
		Model 3	1.54 (1.54-1.55)	<0.001
Perceived Oral Health	Arthritis	Model 1	1.77 (1.75-1.79)	<0.001
	n= 5291	Model 2	1.74 (1.72-1.76)	<0.001
		Model 3	1.69 (1.67-1.71)	<0.001
	W/O arthritis	Model 1	1.88 (1.87-1.89)	<0.001
	n=17,247	Model 2	1.83 (1.82-1.84)	<0.001
		Model 3	1.66 (1.65-1.67)	<0.001

Supplemental Table S3. Association between Sleep Quality with Perceived General and Mental and Oral Health in Participants with Arthritis and Without Arthritis. Model 1: non-adjusted binary logistic regression, Model 2: binary logistic regression adjusted for age and sex, Model 3: binary logistic regression adjusted for age, sex, BMI, smoking status and drinking status. Data presented as odds ratio and 95% confidence intervals; all analysis were weighted. Data considered as statistically significant when p-value ≤ 0.05. W/O= without, OR= Odds Ratio, CI= Confidence Interval, n= number of respondents, ref= reference.
